# A Comparison of Cysteine-Conjugated Nitroxide Spin Labels for Pulse Dipolar EPR Spectroscopy

**DOI:** 10.3390/molecules26247534

**Published:** 2021-12-13

**Authors:** Katrin Ackermann, Alexandra Chapman, Bela E. Bode

**Affiliations:** EaStCHEM School of Chemistry, Biomedical Sciences Research Complex, and Centre of Magnetic Resonance, University of St Andrews, North Haugh, St Andrews KY16 9ST, UK; ka44@st-andrews.ac.uk (K.A.); ac377@st-andrews.ac.uk (A.C.)

**Keywords:** PDS, PELDOR, DEER, nitroxide spin label, Comparative DEER Analyzer, mtsslSuite, MMM

## Abstract

The structure-function and materials paradigms drive research on the understanding of structures and structural heterogeneity of molecules and solids from materials science to structural biology. Functional insights into complex architectures are often gained from a suite of complementary physicochemical methods. In the context of biomacromolecular structures, the use of pulse dipolar electron paramagnetic resonance spectroscopy (PDS) has become increasingly popular. The main interest in PDS is providing long-range nanometre distance distributions that allow for identifying macromolecular topologies, validating structural models and conformational transitions as well as docking of quaternary complexes. Most commonly, cysteines are introduced into protein structures by site-directed mutagenesis and modified site-specifically to a spin-labelled side-chain such as a stable nitroxide radical. In this contribution, we investigate labelling by four different commercial labelling agents that react through different sulfur-specific reactions. Further, the distance distributions obtained are between spin-bearing moieties and need to be related to the protein structure via modelling approaches. Here, we compare two different approaches to modelling these distributions for all four side-chains. The results indicate that there are significant differences in the optimum labelling procedure. All four spin-labels show differences in the ease of labelling and purification. Further challenges arise from the different tether lengths and rotamers of spin-labelled side-chains; both influence the modelling and translation into structures. Our comparison indicates that the spin-label with the shortest tether in the spin-labelled side-group, (bis-(2,2,5,5-Tetramethyl-3-imidazoline-1-oxyl-4-yl) disulfide, may be underappreciated and could increase the resolution of structural studies by PDS if labelling conditions are optimised accordingly.

## 1. Introduction

Research into the functional characteristics of molecules and materials is underpinned by the fundamental dogma that the molecular structure determines properties. Thus, structure determination lies at the core of chemistry, and virtually every undergraduate will learn Bragg’s Law describing X-ray diffraction [1]. The prevalence of structure as a determinant of all properties persists when studying the molecules of life. The fundamental hypothesis that all biomolecular functions are encoded in the structure [2] remains the central dogma of structural biology. The ever-increasing complexity of biological systems under study has been accompanied by a rise in awareness that structural context is of crucial relevance, and integrative structural biology is becoming increasingly important for consolidating information from a variety of methods into a holistic model. Similar approaches allow integrating results from multiple methods for materials characterisation.

X-ray crystallography, especially in its high-throughput forms [3], remains the gold standard for structure determination of crystals from small molecules to solid-state materials. The amorphous nature of polymers and their composites with other components requires a more involved approach. While diverse forms of microscopy (including cryo-electron microscopy), diffraction and scattering (such as small-angle X-ray scattering) and spectroscopic methods provide a plethora of structural data, magnetic resonance can provide structural information with atomic resolution and within native context based on labelling with stable isotopes or exploiting the low natural abundance of unpaired electron spins. Here, we focus on the use of electron paramagnetic resonance (EPR) spectroscopy in determining precise nanometre distances between selected sites in biomolecules [4,5] to validate structural models [6,7] and establish conformational topologies [8,9,10].

For pulse dipolar EPR spectroscopy (PDS) [11,12,13], specific labelling sites within the fold of the protein of choice are subjected to site-directed mutagenesis to establish cysteines at the sites of interest (requiring knockout of other accessible cysteines). These cysteines are site-specifically spin labelled with sulfide-specific labelling reagents to introduce a stable spin bearing moiety, most commonly a nitroxide radical [14,15]. While this arguably provides limited information, yielding merely a single distance distribution per label pair, this can be extremely powerful, especially in combination with complementary methods. Importantly, knowledge of possible spin label conformations is crucial to predict corresponding distance distributions [16,17,18,19,20]. In addition to distance distributions encoding conformational flexibility [21,22], potential weak exchange interactions between the spin centres can be quantified [23,24,25] as well as the number of coupled electron spins interacting in one structural object [12,26,27] and their distribution within nano-confinements [28,29]. Initially informed by a plethora of chemical model systems [30] designed for proof-of-principle and benchmark studies, PDS has allowed significant contributions to the understanding of complex protein systems [24,31]. Illustrative examples are homo-multimeric membrane channels where insights from simulations [32,33] and model systems [34,35] could be translated to significantly improved structural resolution [36,37], and these optimised conditions ultimately yielded functional insights into channel gating [38]. Other examples include the identification of physiologically relevant dimer interfaces in viral proteins [6] and the investigation of the self-assembly of archaeal single-stranded DNA binding proteins [39].

**Scheme 1:**
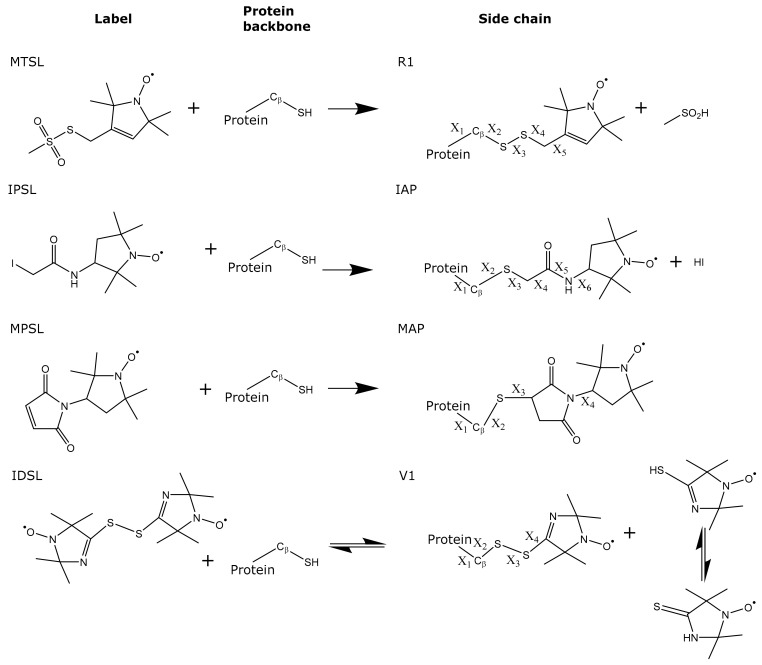
Scheme displaying the four spin labelling compounds (MTSL, IPSL, MPSL and IDSL) used in this study and the resulting cysteine conjugates and leaving groups. Rotatable bonds are indicated (X).

The aim of this study is to investigate four commercially available nitroxide labels (Scheme 1) based on cysteine-mediated conjugation to the protein of interest. Here, we aim to compare labelling efficiency as well as measurement sensitivity and accuracy based on the immunoglobulin-binding B1 domain of group G streptococcal protein G (GB1) that has been extensively used for nitroxide and copper(II) spin labelling [40,41,42,43,44,45]. While numerous detailed characterisations [46,47] and reviews [48,49,50] exist, there is, to our best knowledge, no published study directly comparing these four spin labels. The results indicate a surprisingly large breadth in terms of ease of labelling and purification, and agreement with structural modelling.

## 2. Materials and Methods

### 2.1. Protein Construct and Spin Labelling

The following nitroxide spin labels were employed in this study: MTSL [4,51] ((1-Oxyl-2,2,5,5-tetramethyl-3-pyrroline-3-methyl)methanethiosulfonate; Santa Cruz Biotechnology), IPSL [52,53,54] (3-(2-Iodoacetamido)-2,2,5,5-tetramethyl-1-pyrrolidinyloxy; Sigma-Aldrich), MPSL [55,56,57] (3-Maleimido-2,2,5,5-tetramethyl-1-pyrrolidinyloxy; Santa Cruz Biotechnology), and the biradical IDSL [58,59,60,61] (bis-(2,2,5,5-Tetramethyl-3-imidazoline-1-oxyl-4-yl)disulfide; Noxygen). MTSL and IDSL stock solutions were prepared at 100 mM concentration in dimethyl sulfoxide (DMSO) and stored at −80 °C; IDSL required sonication for full dissolution. An MPSL stock solution was prepared at a 100 mM concentration in 100% ethanol; an IPSL stock solution was prepared at a 77 mM concentration in 100% methanol. MPSL and IPSL stock solutions were aliquoted at 1 mg label per aliquot (40 and 42 μL per aliquot for IPSL and MPSL, respectively), dried in a SpeedVac and stored at −20 °C.

A double-cysteine mutant (I6C/K28C) of the immunoglobulin-binding B1 domain of group G streptococcal protein G (GB1) was used as the model protein. In this mutant, one cysteine is introduced into an α-helix (K28C) while the second cysteine is located in a β-sheet (I6C) [41]. Expression and purification were performed as described previously [40,43]. 12 mg of GB1 I6C/K28C in 2 mL of phosphate buffer (150 mM NaCl, 42.4 mM Na_2_HPO_4_, 7.6 mM KH_2_PO_4_, pH 7.4) were freshly reduced with dithiothreitol (DTT) using a 5-fold DTT concentration per cysteine (10-fold per protein molecule) overnight at 4 °C. DTT was removed using a desalting PD10 column, and the eluted protein solution was split into 8 equal parts for labelling.

Two labelling reactions per spin label were set up, each adding a 3-fold molar concentration of label per cysteine (6-fold per protein molecule). MTSL and IDSL were added from the DMSO stock, while MPSL and IPSL were redissolved in ethanol and methanol before use, respectively. One sample set was kept at room temperature in the dark for 2 h while the second set was kept in the dark at 4 °C overnight. After their respective incubation periods, aliquots were taken and immediately frozen before submission to mass spectrometry (MALDI-TOF) using the in-house facility to confirm labelling. The residual free label was removed via PD10 columns, and aliquots for spin counting to determine labelling efficiencies via continuous wave (CW) EPR, aliquots for mass spectrometry (MALDI-TOF) of the purified samples, as well as the remaining samples, were frozen until use.

For IDSL, an additional labelling reaction was performed using a 20-fold molar concentration of the label per cysteine, with incubation overnight at 4 °C.

### 2.2. Continuous Wave (CW) EPR Spectroscopy

Room-temperature CW EPR measurements to determine labelling efficiencies were performed using a Bruker EMX 10/12 spectrometer equipped with an ELEXSYS Super Hi-Q resonator at an operating frequency of ~9.9 GHz (X-band) with 100 kHz modulation. 50 μL samples in micro capillaries (Brand; one end flame-sealed) were recorded using a 120 G field sweep centred at 3445 G, a time constant of 20.48 ms, a conversion time of 20.10 ms and 2048 points resolution. An attenuation of 20 dB (2 mW power), 50 dB receiver gain and a modulation amplitude of 0.7 G were used for all samples. GB1 samples were measured at a ~50 μΜ protein (~100 μΜ spin) concentration, and double integrals (corrected for the actual protein concentration and the number of scans) were compared to 100 μΜ MTSL as a standard.

### 2.3. Pulse Dipolar EPR—Sample Preparation and Measurement

EPR samples from the overnight incubation for each spin label were prepared at a 24 μM final protein concentration with 50% ethylene glycol for cryoprotection. For buffer exchange into the deuterated solvent, 100 μL of each protonated labelled sample were freeze-dried and reconstituted in 100 μL D_2_O. Samples were prepared at a 24 μM final protein concentration with 50% fully deuterated ethylene glycol for cryoprotection. All samples had a final volume of 65 μL and were transferred to 3 mm quartz EPR tubes, which were immediately frozen by immersion into liquid nitrogen.

PDS experiments were performed at Q-band frequency (34 GHz) operating on a Bruker ELEXSYS E580 spectrometer with a 3 mm cylindrical resonator (ER 5106QT-2w in TE012 mode) using a second frequency option (E580-400U). The temperature was controlled via a cryogen-free variable temperature cryostat (Cryogenic Ltd.) operating in the 3.5 to 300 K temperature range. Pulses were amplified by a pulse travelling wave tube (TWT) amplifier (Applied Systems Engineering) with a nominal output of 150 W.

Specifically, pulsed electron–electron double resonance (PELDOR/DEER) experiments were performed with the 4-pulse DEER [13,62,63] pulse sequence (π/2(ν_A_) − τ_1_ − π(ν_A_) − (τ_1_ + t) − π(ν_B_) − (τ_2_ − t) − π(ν_A_) − τ_2_ − echo) at 50 K as described previously, [6] with a frequency offset (pump—detection frequency) of +80 MHz (~3 mT). The shot repetition time (SRT) was set to 4 ms (deuterated samples) or 3 to 4.5 ms (protonated samples); τ_1_ was set to 380 ns, and τ_2_ was set to 8000 ns for the deuterated samples and to 2400 ns for the protonated samples apart from the IDSL-labelled GB1 sample where 3200 ns were used to allow sufficient resolution for the detection of longer distances. Pulse lengths were 16 and 32 ns for π/2 and π detection, and 12 ns for the ELDOR pump π pulse. The pump pulse was placed on the resonance frequency of the resonator and applied to the maximum of the nitroxide field-swept spectrum.

PELDOR data were subjected to the Comparative DEER Analyzer (CDA) within DeerAnalysis2021b [64] for unbiased data processing and analysis according to recent recommendations [65], employing DEERNet [66] neural network processing and DeerLab [67] Tikhonov regularisation. Full reports of the CDA analysis are provided in the supplementary information.

### 2.4. Modelling

Distance distributions were modelled based on the I6H/N8H/K28H/Q32H construct (PDB ID: 4WH4) [41]; histidine residues at positions 6 and 28 were mutated to cysteine residues, while histidine residues at positions 8 and 32 were mutated to asparagine and glutamine residues, respectively.

Modelling was performed using the MATLAB plugin MMM 2018 [19] under ambient (298 K) and cryogenic (175 K) temperature conditions and compared with modelling using the mtsslWizard [17] within the mtsslSuite [68] server-based modelling software (Table 1) using ‘tight’ (vdW-restraint 0 clashes, 3.4 Å cutoff) and ‘loose’ (vdW-restraint 5 clashes, 2.5 Å cutoff) settings. Cartoon structural representations of spin-labelled GB1 were generated using Pymol [69].

## 3. Results and Discussion

### 3.1. Spin Labelling

A deliberately small ratio of spin label to cysteine of 3 to 1 was chosen to allow for assessing differences in the ease of labelling for the different nitroxide labels. In addition, two different incubation conditions were tested for each label, a quick labelling reaction of 2 h at room temperature and an overnight labelling reaction at 4 °C. Successful labelling and labelling efficiencies were determined using mass spectrometry and continuous wave (CW) EPR spectroscopy, respectively.

#### 3.1.1. CW EPR

**Figure 1:**
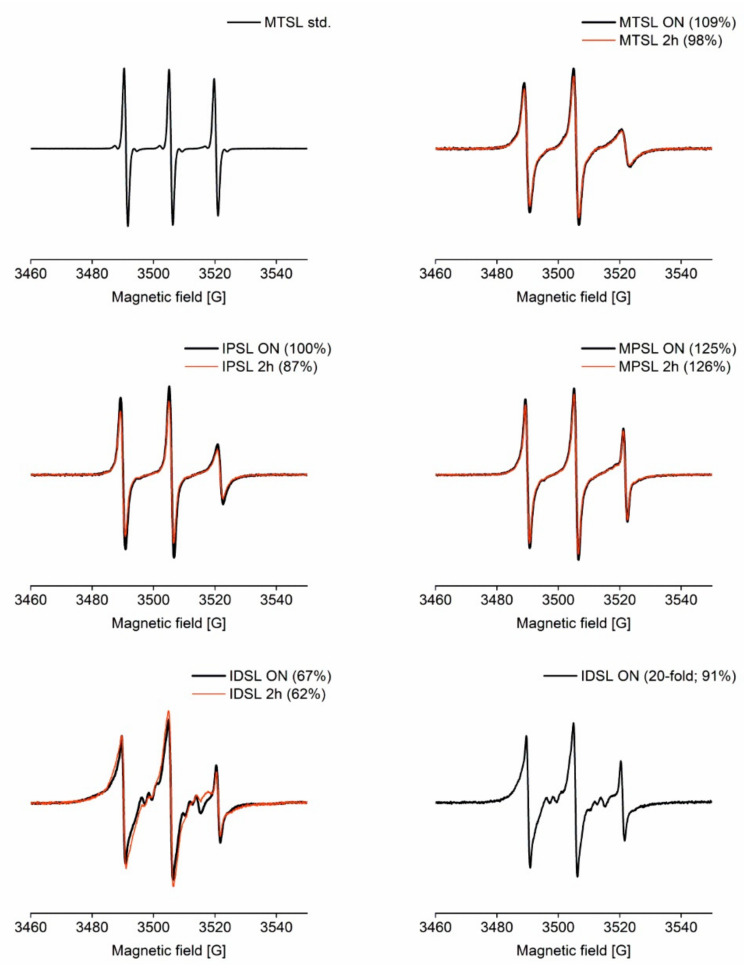
Continuous wave (CW) EPR spectra for GB1 labelled with MTSL, IPSL, MPSL and IDSL and respective labelling efficiencies relative to MTSL standard. All spectra were recorded with the same experimental parameters and normalised to the number of scans. 2 h = two hours incubation time; ON = overnight incubation time. For IDSL-labelled GB1, due to the low labelling efficiencies for a 3-fold label concentration, an additional labelling experiment was performed at a 20-fold label concentration incubating overnight.

Individual CW EPR spectra are shown in Figure 1, and a summary of labelling efficiencies is given in Table 2. MTSL labelling efficiency was around 100% after just two hours of labelling. MPSL labelling was determined at about 125%, indicating more label present than available cysteines, already after two hours. The sharp component (especially visible in the high-field line) in MPSL-labelled GB1 spectra suggests that some free label might be present in the samples despite the PD10 column used to remove the free label. This would explain the determined labelling efficiency of well above 100% and indicates purification protocols that were empirically optimised for MTSL may not be sufficient for MPSL. For IPSL, quantitative labelling was achieved after the overnight incubation. The biradical IDSL is attached by substituting one sulfide of its disulfide bond. In contrast to substitutions with good leaving groups (MTSL and IPSL) or addition reactions (MPSL), this disulfide exchange has an equilibrium constant closer to unity, thus incomplete labelling and free dimeric label are very likely. It should be noted that the free thiolate released as leaving group can attack another disulfide bond and this can, at least in theory, result in the complete scrambling of disulfide bonds that could also entail disulfide-linked protein dimers, if steric demand around the cysteine residues permits. IDSL did not yield more than two-thirds of labelling efficiency even after the overnight incubation time. Therefore, a second overnight labelling reaction using a 20-fold concentration of IDSL with respect to cysteine was performed, which yielded above 90% efficiency. Additional lines in IDSL-labelled GB1 spectra are attributed to the intact (free) label [60].

CW data suggest that MTSL, IPSL and MPSL can provide quantitative labelling at relatively small (here: 3:1) label-to-cysteine ratios for both secondary structure elements, α-helix and β-sheet. It should be noted that both labelling sites in the GB1 construct are easily accessible, thus higher ratios might be required if sites are buried. MTSL and IPSL could easily be removed using a PD10 desalting column, while MPSL labelling might need additional chromatographic steps to remove the residual free label. IDSL, presumably due to the equilibrium reaction, was shown to require a higher excess of the label to approach quantitative labelling of the cysteine residues.

#### 3.1.2. Mass Spectrometry

Samples were analysed by MALDI-TOF after 2 h and overnight incubation time and a 3:1 label-to-cysteine ratio confirming successful spin labelling before the removal of excess free label. Since overnight reactions generally showed better labelling, these were taken forward for PDS and the purified samples were re-analysed by MALDI-TOF, showing excellent agreement with results from the unpurified labelling reactions. In the case of IDSL-labelled GB1, MALDI-TOF was also performed after overnight reaction with 20-fold IDSL. Details and individual mass spectrometry results are shown in the supplementary information (Figures S1–S10). Overall, MALDI-TOF spectra are in line with the results obtained from CW EPR; although for IPSL- and especially IDSL-labelled GB1, less of the fully labelled protein is seen with MALDI-TOF than would be expected from CW EPR. This could be due to residual free label (not all labels attached to the protein), label-specific differences in the ionisation for labelled and unlabelled protein or the laser could lead to partial label detachment. Interestingly, upon measuring the second distance in PELDOR for IDSL-labelled GB1 (see below), MALDI could also confirm the presence of a small amount of a species with a mass corresponding to a GB1 dimer, which was no longer present in the sample with a 20-fold ratio of label-to-cysteine. This highlights the need for a larger excess of the IDSL label—not only to drive the equilibrium towards quantitative cysteine labelling, but also to avoid significant equilibrium concentrations of the disulfide-linked protein dimer formed by thiolate exchanges.

### 3.2. PDS Distance Measurements (PELDOR/DEER) and Comparison to Modelling

**Figure 2:**
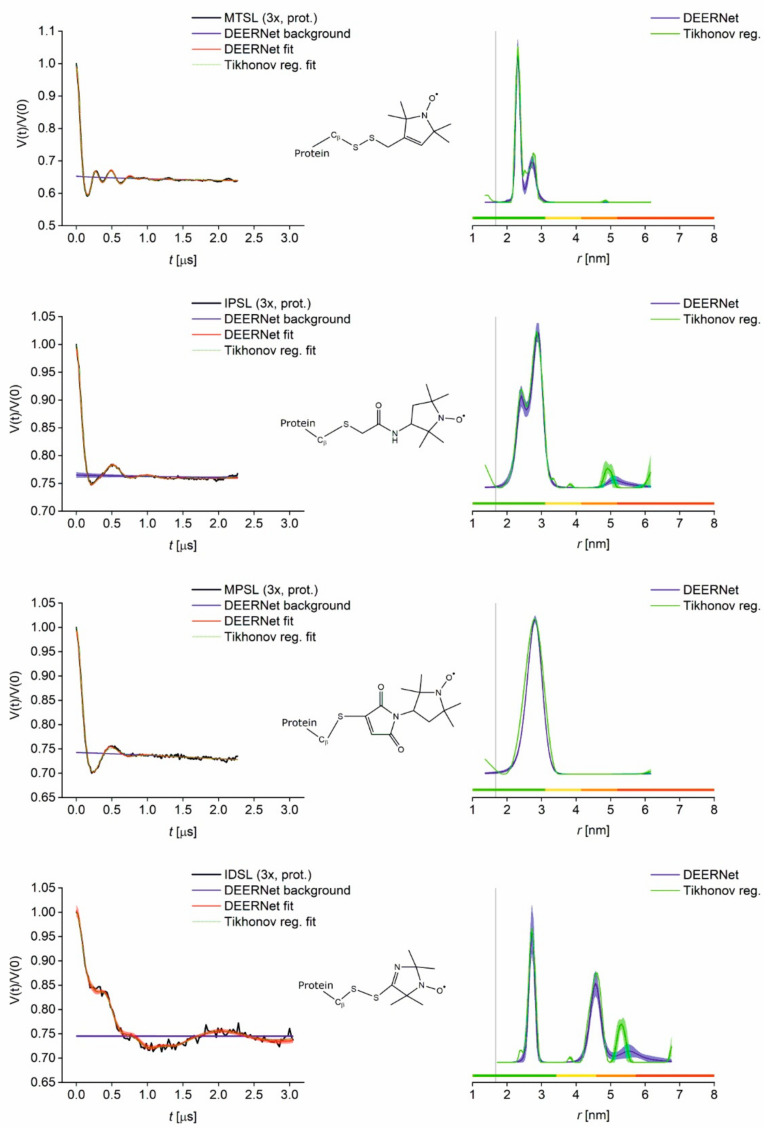
PELDOR results for protonated GB1 I6C/K28C labelled with MTSL, IPSL, MPSL and IDSL. Left: Superposition of primary data (black) with Tikhonov regularisation fit (green) and DEERNet separated background (blue) and fit (red) with uncertainty bounds. Right: Superposition of distance distributions obtained from DEERNet (blue) and Tikhonov regularisation (green) with uncertainty bands. Vertical lines indicate the C_β_-C_β_ distance 6C–28C (1.67 nm). Colour bars represent reliability ranges (green: Shape reliable; yellow: Mean and width reliable; orange: Mean reliable; red: No quantification possible).

Initially, protonated samples from the overnight labelling reaction were prepared for PDS after removal of the free spin label. We rationalised that echo dephasing (*T_m_*) would be sufficient to resolve the expected short distance (below 3 nm) in the GB1 I6C/K28C construct. Distance distributions obtained from PDS (PELDOR/DEER) primary data on the protonated samples are shown in Figure 2.

MTSL and, to a lesser extent, IPSL labelling results suggest a bimodal distance distribution within the range of 2–3 nm, while MPSL and IDSL labelling results do not. An interesting finding is the appearance of a larger distance (~4.5 nm) for IDSL-labelled GB1, further supporting the hypothesis of dimer formation based on label chemistry and mass spectrometry, which is further discussed below. However, distance resolution is not sufficient in the protonated samples, therefore the second set of samples was prepared with reconstitution of the freeze-dried protein into D_2_O and a fully deuterated cryoprotectant. A comparison of respective refocused echo decay experiments demonstrating the gain in the distance resolution with deuteration is given in Figure S13. In addition, IDSL labelling was repeated using a much larger excess of the label (ratio 20:1) to assess changes in the labelling efficiency and the presence of the disulfide-linked protein dimer.

**Figure 3:**
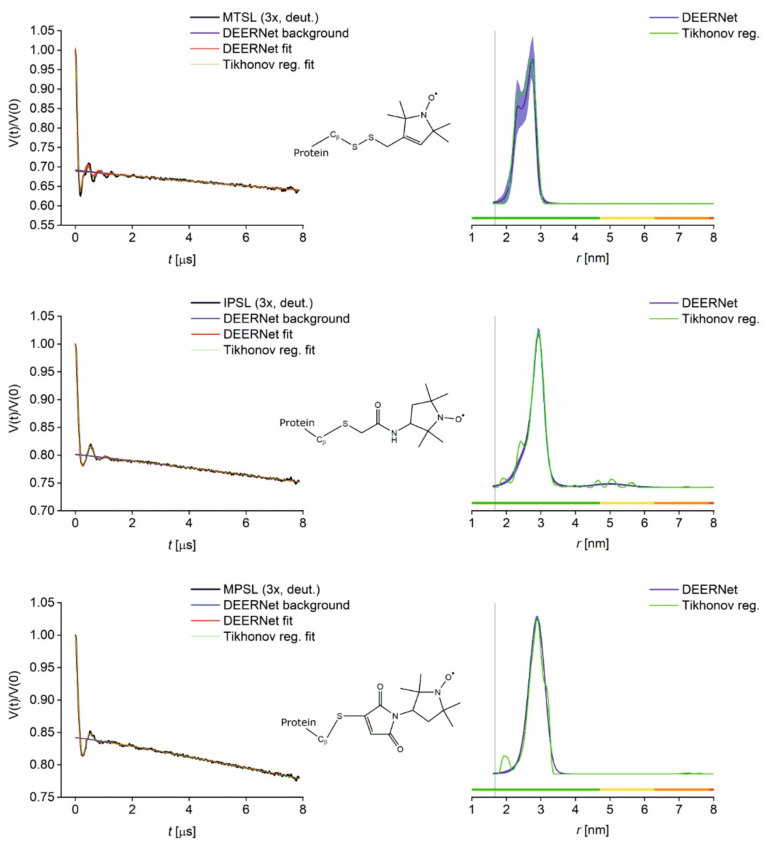
PELDOR results for GB1 I6C/K28C labelled with MTSL, IPSL and MPSL and buffer exchanged into deuterated solvent. Left: Superposition of primary data (black) with Tikhonov regularisation fit (green) and DEERNet separated background (blue) and fit (red) with uncertainty bounds. Right: Superposition of distance distributions obtained from DEERNet (blue) and Tikhonov regularisation (green) with uncertainty bands. Vertical lines indicate the C_β_-C_β_ distance 6C–28C (1.67 nm). Colour bars represent reliability ranges (green: Shape reliable; yellow: Mean and width reliable; orange: Mean reliable; red: No quantification possible).

**Figure 4:**
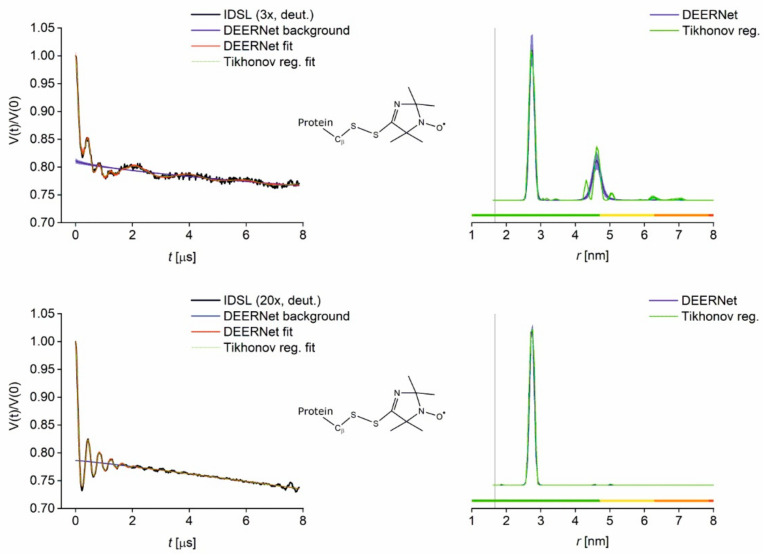
PELDOR results for GB1 I6C/K28C labelled with IDSL at 3:1 and 20:1 label-to-cysteine ratio and buffer exchanged into deuterated solvent. Left: Superposition of primary data (black) with Tikhonov regularisation fit (green) and DEERNet separated background (blue) and fit (red) with uncertainty bounds. Right: Superposition of distance distributions obtained from DEERNet (blue) and Tikhonov regularisation (green) with uncertainty bands. Vertical lines indicate the C_β_-C_β_ distance 6C–28C (1.67 nm). Colour bars represent reliability ranges (green: Shape reliable; yellow: Mean and width reliable; orange: Mean reliable; red: No quantification possible).

Distance distributions obtained from PDS (PELDOR/DEER) primary data on the deuterated samples are shown below (Figure 3 and Figure 4), and a superposition of distributions obtained from protonated and deuterated samples is given Figure S12. MTSL labelling still suggests a bimodal distance distribution, but to a lesser extent than observed for the protonated sample. This bimodality with MTSL has been observed previously [40] and can be rationalised with additional label conformations. Another potential explanation would be the presence of different conformational states of the protein itself. However, GB1 is not known to exist in a conformational equilibrium but is a very rigid small protein model. If there were additional protein conformations, these would be expected to also be visible with the other labels, which is not the case.

For IDSL-labelled GB1, the larger distance (~4.5 nm) at the 3:1 label-to-cysteine ratio is confirmed with the deuterated sample with a sufficient distance resolution. If the thiolate exchange is dynamic, then a certain fraction of the disulfide-bridged protein dimer will equilibrate based on its proportion in the mixture. A disulfide-bridged GB1 protein dimer will still have one cysteine per monomer available for labelling. Considering a distance of just over 2.5 nm for IDSL-labelled GB1 and a distance of 1.7 nm between C_β_ atoms of the labelled residues, a disulfide-bridged dimer of singly IDSL-labelled GB1 monomers will have a distance shorter than two times 2.5 nm but substantially longer than two times 1.7 nm, in good agreement with the 4.5 nm observed. SDS-PAGE for spin-labelled GB1 I6C/K28C with non-reduced samples to preserve disulfide linkages demonstrates that disulfides do indeed form for IDSL at the 3:1 label-to-cysteine ratio (see Figure S11). As expected, this dimer peak vanished after increasing the amount of IDSL label to a 20:1 ratio, indicating the equilibrium labelling reaction can be driven towards quantitative binding by a larger excess of the label. In this case, a labelling efficiency of >90% was determined using CW EPR. Notably, due to the shortest linker of all labels tested, the IDSL provides the highest precision with significantly narrower distributions, similar to those observed for Cu^II^-labelling of double-histidine sites [41,43].

As our samples may contain free label, it is important to note that the residual free label will add to the unmodulated part of the echo, thereby reducing modulation depth. However, it should not alter the distance distribution or influence the overall signal-to-noise. Unspecific labelling (i.e., with the label attached to the protein at a non-cysteine residue) might lead to added modulation depth (and potentially multi-spin effects), and additional distances affecting the overall distance distribution. We did not observe these effects in our PDS data nor indications of unspecific labelling from mass spectrometry.

**Figure 5:**
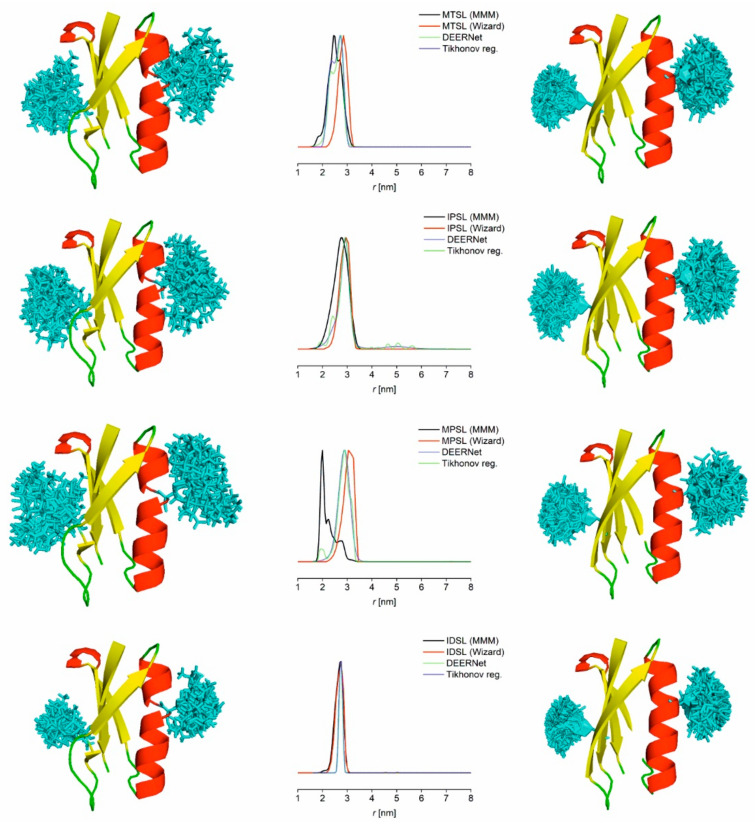
Modelled distance distributions for GB1 I6C/K28C labelled with MTSL, IPSL, MPSL and IDSL. Left: Cartoon representation of GB1 and modelled spin label conformations (cyan sticks) as obtained from MMM. Middle: Superposition of predicted and experimental (buffer exchanged into deuterated solvent, 3:1 ratio except IDSL 20:1 ratio) distance distributions. Right: Cartoon representation of GB1 and modelled spin label conformations (cyan sticks) as obtained from mtsslWizard.

Modelling of expected distance distributions for the four nitroxide labels was performed using MMM and mtsslWizard; overlays of the resulting simulated and experimental distributions are shown in Figure 5 for recommended settings (MMM at ambient temperature, mtsslWizard with ‘tight’ vdW-restraint setting) and in Figure S14 with additional settings (MMM at cryogenic temperature, mtsslWizard with ‘loose’ vdW-restraint setting). Overall, consistent models were obtained. The bimodal distance distribution observed with the protonated sample of MTSL-labelled GB1 is in contrast to modelling results. This difference could be rationalised with an additional label conformation induced by the interaction with the protein surface escaping both modelling approaches [16,20]. Interestingly, this seems to be much less pronounced in the deuterated sample even though all other conditions are nominally identical. For IPSL-labelled GB1, the modelled distributions matched well with the experimentally obtained distribution, while for MPSL-labelled GB1, some deviation between the two modelling approaches was observed, with the mtsslWizard model matching experimental distributions more closely. As seen in Figure 5, the spin-labelled side-chain of IDSL-labelled GB1 occupies the most restricted rotameric space of all four labels tested. Notably, experimental distance distributions were even narrower than the simulated ones. Expectedly, the distance corresponding to the disulfide-bridged GB1 dimer could not be modelled as it is not present in the coordinate file.

We further investigated the agreement between the two modelling approaches (MMM and mtsslWizard) and their agreement with experimental distance distributions obtained from two different processing methods (neuronal network analysis and Tikhonov regularisation). Comparing deviations of the distributions obtained from protonated and deuterated buffer samples reveals that the agreement between different modelling approaches and the corresponding experiments is not much worse than between different samples (Table 3). MTSL labelling leads to different populations of conformers and IDSL labelling leads to a different population of dimers dominating the rmsd between experiments. For the latter, both modelling approaches are in excellent agreement, but the width of the distribution is found to be narrower than modelled and this dominates the rmsd. Interestingly, the model deviation is worst for MPSL-labelled GB1, where this seems largely down to modelled short-distance conformers in MMM at 298 K that do not manifest in the experimental distribution with the agreement between experiment and mtsslWizard being significantly better. Here, the agreement between experiments for protonated and deuterated samples is best for all labels. Interestingly, MMM at 175 K substantially shifts the modelled distance distribution for MPSL-labelled GB1, giving much better agreement than at ambient temperatures, while ‘loose’ settings for the mtsslWizard result in broadened and slightly shortened distributions for all labels (Figure S14). In contrast, for MTSL-labelled GB1, both models agree better with the experiment than with each other. While there are significant differences between protonated and deuterated samples, MMM agrees much better with the latter than mtsslWizard. On the other hand, the distance distributions for IPSL-labelled GB1 are predicted remarkably well, especially by mtsslWizard. Finally, it is obvious that the prediction of spin-label conformations remains problematic for highly accurate and precise distance measurements, and although IDSL and IPSL labelling appear more robust in this example, it remains to be seen if this holds for other scenarios. However, these results reveal that there is significant promise in systematic comparisons between labels both in silico and in experiments to reveal systematic trends with respect to the reliability of predictions and specific advantages and disadvantages of individual labels.

## 4. Conclusions and Outlook

While MTSL is arguably the current ‘work-horse’ and best-established nitroxide spin label for protein labelling, this study shows that other commercially available cysteine-reactive nitroxide labels are attractive alternatives. Both MPSL and IPSL are supposedly less prone to suffering from cleavage in reducing environments and thus might be better options for more native environments such as in-cell studies, presuming the samples can be frozen before the nitroxide itself is reduced. In our hands, even at a low label-to-cysteine ratio, excess MPSL was not fully removed using a simple PD10 desalting column, suggesting that this label is more demanding in downstream purification steps. In contrast, IPSL delivered quantitative labelling after overnight incubation at the same low excess and was fully removed in the PD10 clean-up step. A very interesting alternative spin label is the biradical IDSL, which has the most restricted rotameric space of all labels compared in this study. However, it requires a large excess of the label to achieve near quantitative labelling. PELDOR results agreed well with modelling based on the crystal structure for all four labels and the differences between modelling approaches and between analysis methods were generally similar to the deviations between the models and experiments. Notably, distance distributions for IDSL-labelled GB1 exhibited significantly enhanced precision due to the short linker that is truer to the protein backbone. This makes IDSL particularly attractive to investigate small conformational changes requiring a high precision with narrow distributions. It will be interesting to further explore the specific advantages of the individual labels for distinct applications.

The research data underpinning this publication can be accessed at https://doi.org/10.17630/70739623-1bb5-40e6-b326-5fff1e22c289 [70].

## Figures and Tables

**Table 1:** List of nitroxide spin labels used in this study and respective names of modifications in modelling software.

Spin Label Name This Study	Side-Chain in MMM	Side-Chain in MtsslSuite
MTSL	MTSL	R1
IPSL	IA-Proxyl	Proxyl
MPSL	MA-Proxyl	malR1
IDSL	V1	sR1

**Table 2:** Summary of determined labelling efficiencies per cysteine relative to MTSL standard.

Incubation	MTSL	IPSL	MPSL	IDSL
2 h, 3-fold	98%	87%	126%	62%
ON, 3-fold	109%	100%	125%	67%
ON, 20-fold	-	-	-	91%

**Table 3:** Root-mean-square-deviations of intensity-normalised distance distributions based on various experiments and modelling approaches.

	Wizard/MMM	Deuterated/Protonated		DEERNet		Regularised	
	RMSD	DEERNet	Regularised	Deuterated/Wizard	Deuterated/MMM	Deuterated/Wizard	Deuterated/MMM
MTSL	0.1363	0.1118	0.1161	0.1165	0.0459	0.1237	0.0421
IPSL	0.0803	0.0568	0.0628	0.0311	0.0750	0.0340	0.0732
MPSL	0.1866	0.0432	0.0613	0.0981	0.1741	0.0917	0.1624
IDSL	0.0156	0.0907	0.1099	0.0771	0.0819	0.0714	0.0769

For IDSL values from the deuterated sample using the 20:1 label-to-cysteine ratio were used. RMSD = root mean square deviation; Wizard = mtsslWizard; Regularised = Tikhonov regularisation.

## Data Availability

Digital data underpinning the results presented in this manuscript is freely available [70].

## Supplementary Materials

The following are available online, Mass spectrometry data (Figures S1–S10); SDS-PAGE gel of spin-labelled GB1 6C/28C (Figure S11); superposition of distance distributions of protonated and deuterated samples (Figure S12); refocused echo decay (Hahn echo) experiments (Figure S13); additional modelling (Figure S14), and full CDA reports.

## Sample Availability

Samples of the compounds are not available from the authors.
