# Supplementary material for: A Comparison of Cysteine-Conjugated Nitroxide Spin Labels for Pulse Dipolar EPR Spectroscopy
Source: Molecules. 2021 Dec 13;26(24):7534. doi: 10.3390/molecules26247534 (PMC8706713; doi:10.3390/molecules26247534)
Supplement: Supplementary file 1 [file molecules-26-07534-s001.zip › molecules-1463358-supplementary.pdf]

Supplementary information

A comparison of cysteine-conjugated nitroxide spin labels for pulse dipolar EPR spectroscopy

Katrin Ackermann, Alexandra Chapman, Bela E. Bode*

EaStCHEM School of Chemistry, Biomedical Sciences Research Complex, and Centre of Magnetic Resonance, University of St Andrews, North Haugh, St Andrews, KY16 9ST, Scotland

Content:

Mass spectrometry	page 2
SDS-PAGE gel of spin-labelled GB1 6C/28C	page 10
Superposition of distance distributions of protonated and deuterated samples	page 11
Refocused echo decay (Hahn echo) experiments	page 12
Additional modelling	page 13
CDA reports	page 14

Mass spectrometry

Mass spectrometry was performed in-house using a Sciex Matrix Assisted Laser Desorption/Ionisation (MALDI) TOF/TOF 4800 mass-spectrometer, with samples crystallised using a matrix of α-cyano-4-hydroxycinnamic acid. The MALDI analysis was calibrated externally with Ubiquitin (8585 and 4283m/z) prior to analysis; the acceptable error in mass is given as ± 3 Da for this mass range and signal-to-noise. Mass differences expected are +184 for singly MTSL-labelled, +197 for singly IPSL-labelled, +237 for singly MPSL-labelled, and +171 for singly IDSL-labelled.

In the first set, control (unlabelled) and labelled GB1 I6C/K28C were prepared at 20 µM concentration in phosphate buffer (150 mM NaCl, 42.4 mM Na_2HPO_4, 7.6 mM KH_2PO_4, pH 7.4) and immediately frozen to avoid the labelling reaction to continue. Individual spectra are shown below.

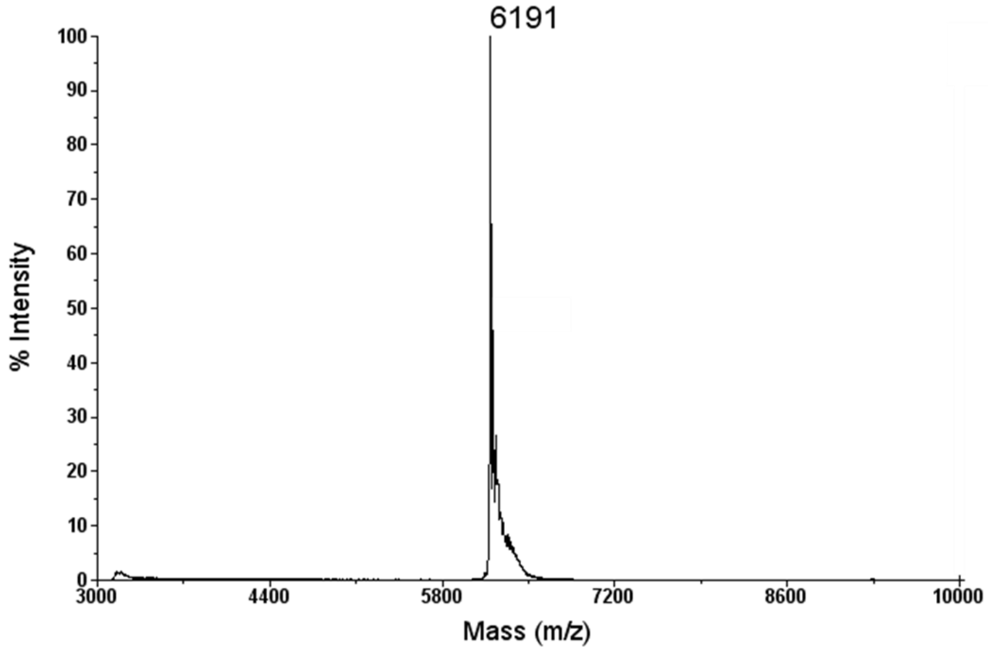

Figure S1. GB1 I6C/K28C control sample (unlabelled). Expected molecular weight: 6188 Da.

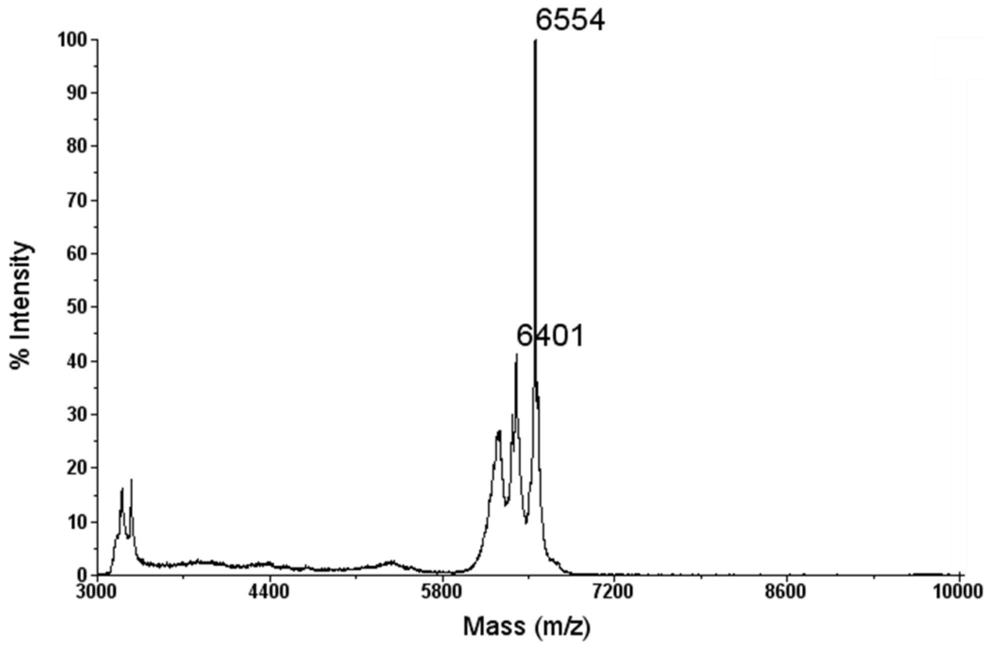

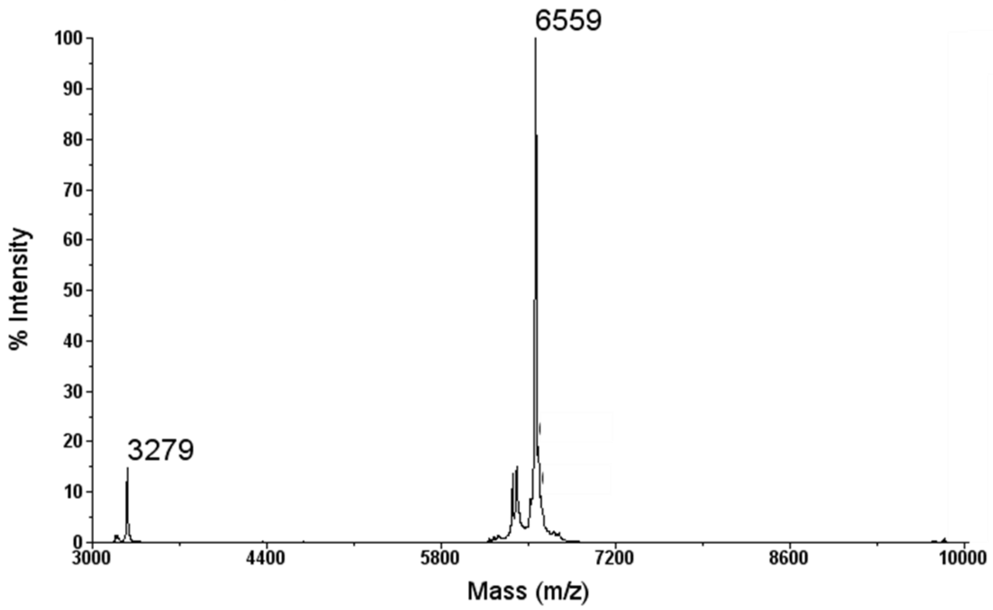

Figure S2. GB1 I6C/K28C sample labelled with MTSL for 2 hours (top) and overnight (bottom). Expected molecular weight: 6556 Da (doubly MTSL-labelled); 6372 Da (singly MTSL-labelled).

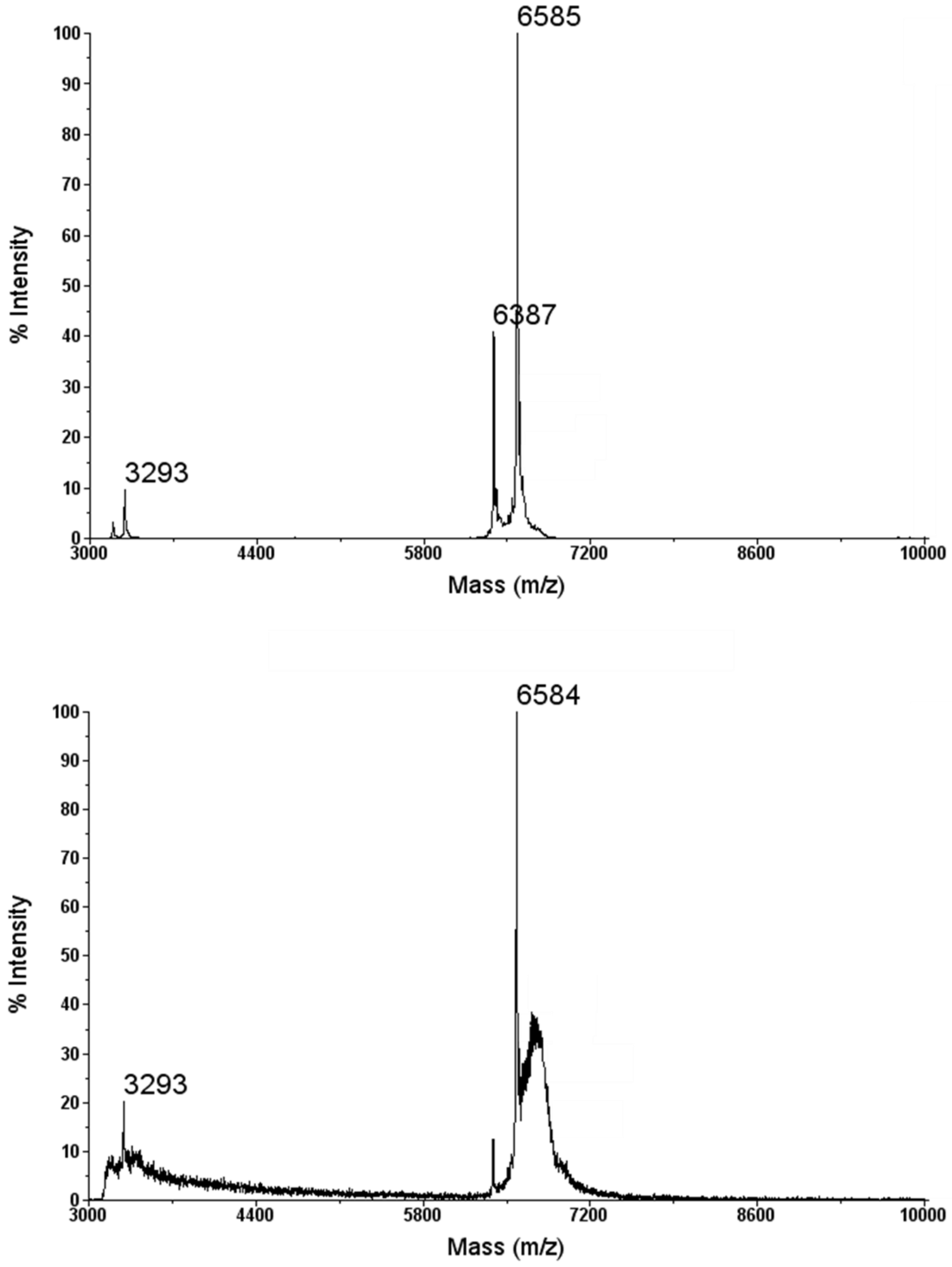

Figure S3. GB1 I6C/K28C sample labelled with IPSL for 2 hours (top) and overnight (bottom). Expected molecular weight: 6582 Da (doubly IPSL-labelled); 6385 Da (singly IPSL-labelled).

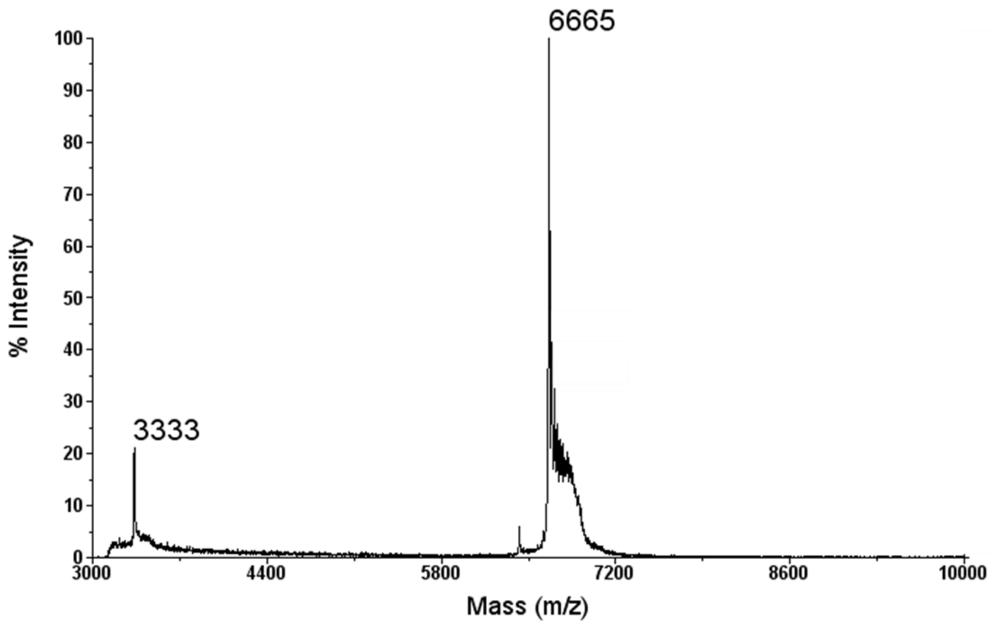

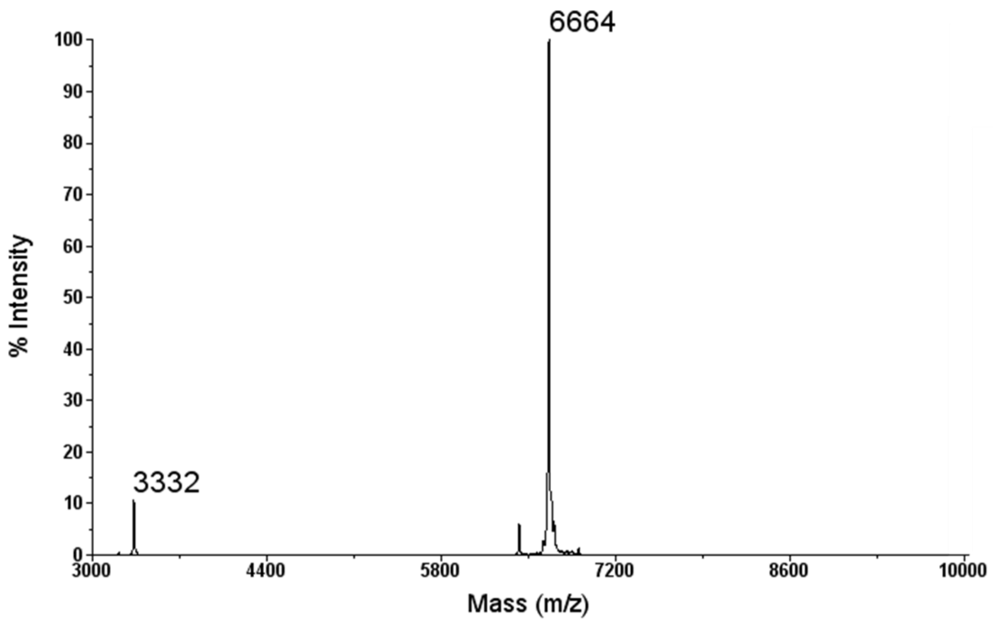

Figure S4. GB1 I6C/K28C sample labelled with MPSL for 2 hours (top) and overnight (bottom). Expected molecular weight: 6662 Da (doubly MPSL-labelled); 6425 Da (singly MPSL-labelled).

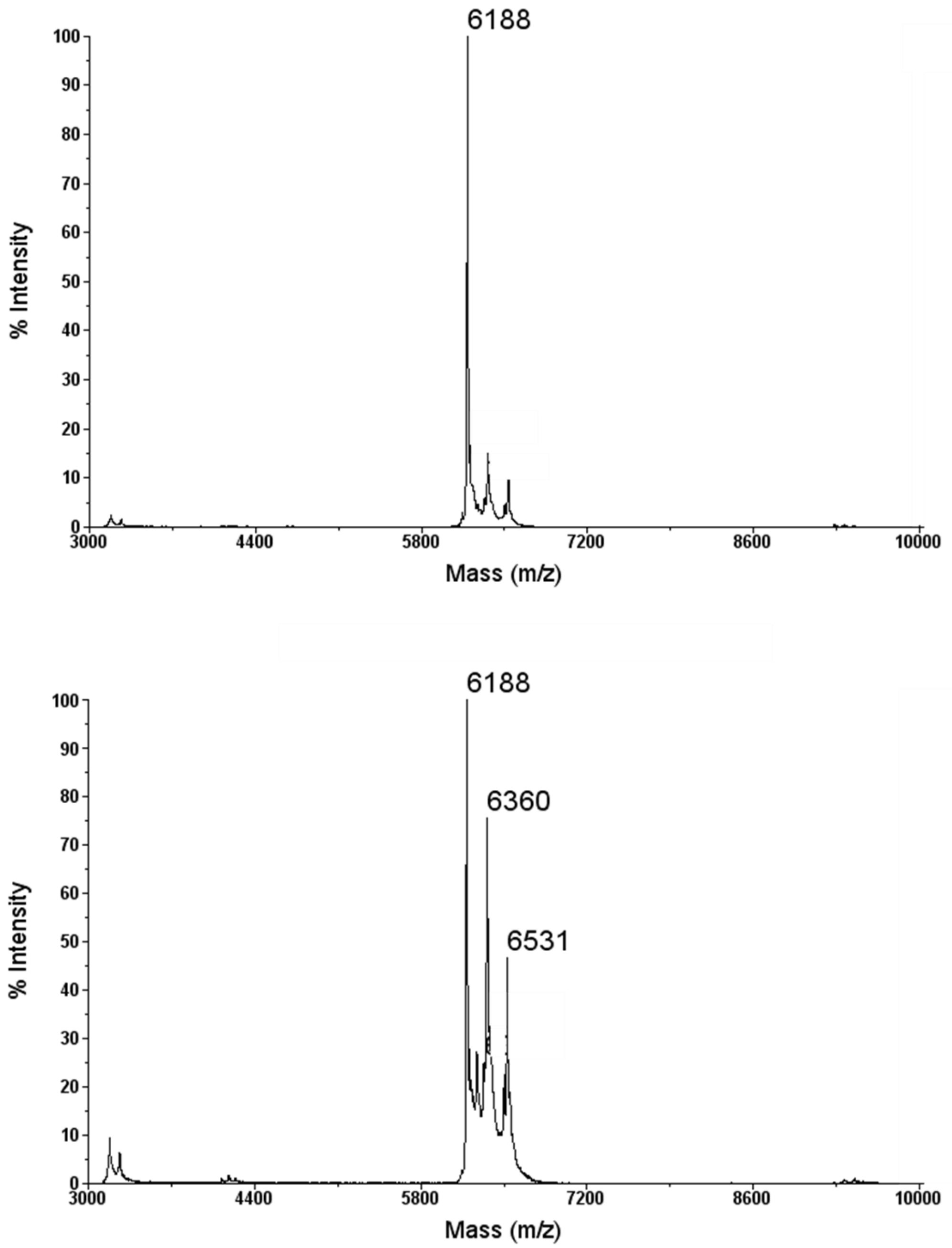

Figure S5. GB1 I6C/K28C sample labelled with IDSL for 2 hours (top) and overnight (bottom). Expected molecular weight: 6530 Da (doubly IDSL-labelled); 6359 Da (singly IDSL-labelled).

In the second set, samples were prepared as above, with the difference that the spin labels had been removed via PD10 column. For IDSL-labelled GB1, an additional sample from 20-fold label-to-cysteine labelling was prepared for MALDI. All samples were processed to the range of 3,000 to 20,000 Da to include the dimer mass.

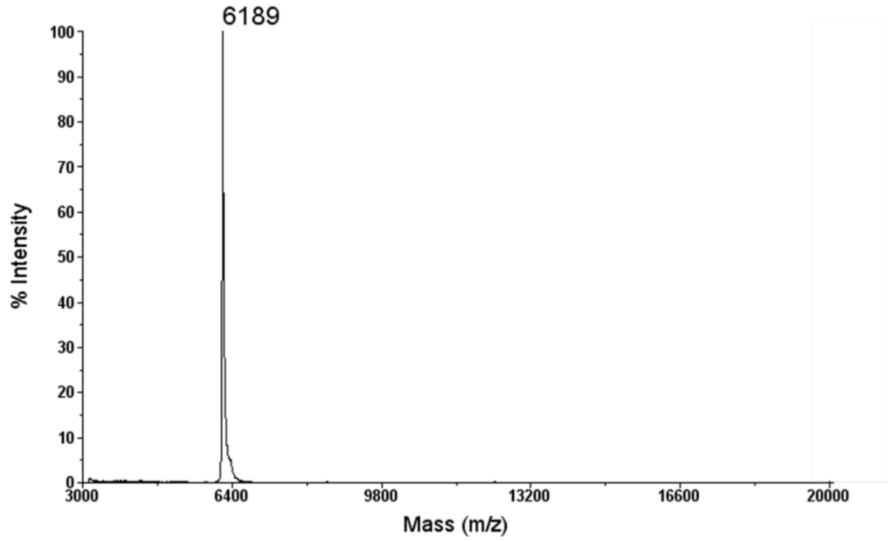

Figure S6. MALDI-TOF spectra with 3 to 20 kDa range after removal of free label. GB1 I6C/K28C control sample (unlabelled). Expected molecular weight: 6188 Da.

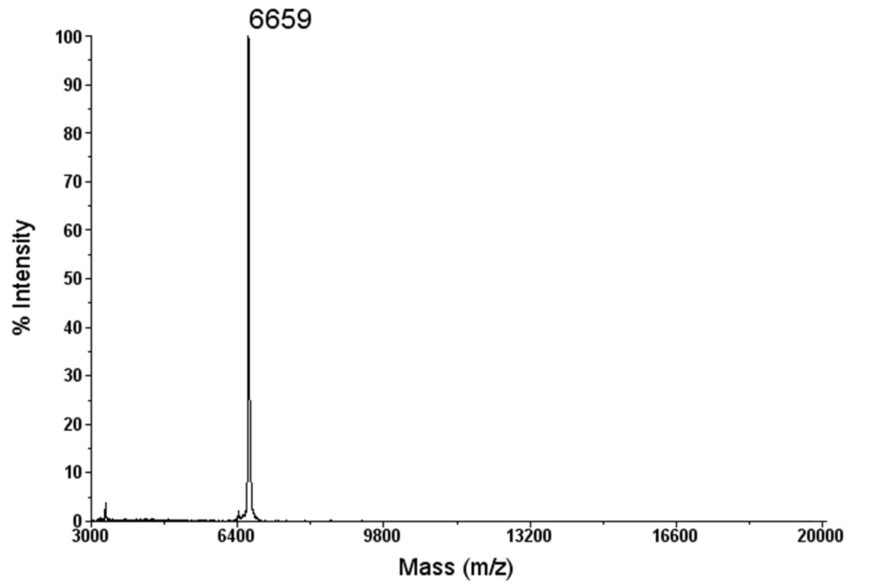

Figure S7. MALDI-TOF spectra with 3 to 20 kDa range after removal of free label. GB1 I6C/K28C sample labelled with MTSL overnight. Expected molecular weight: 6556 Da (doubly MTSL-labelled); 6372 Da (singly MTSL-labelled).

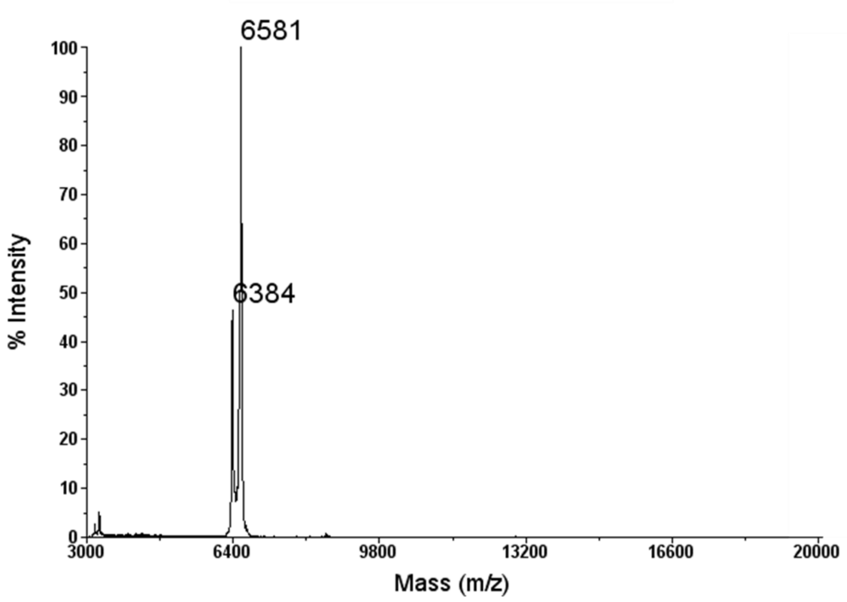

Figure S8. MALDI-TOF spectra with 3 to 20 kDa range after removal of free label. GB1 I6C/K28C sample labelled with IPSL overnight. Expected molecular weight: 6582 Da (doubly IPSL-labelled); 6385 Da (singly IPSL-labelled).

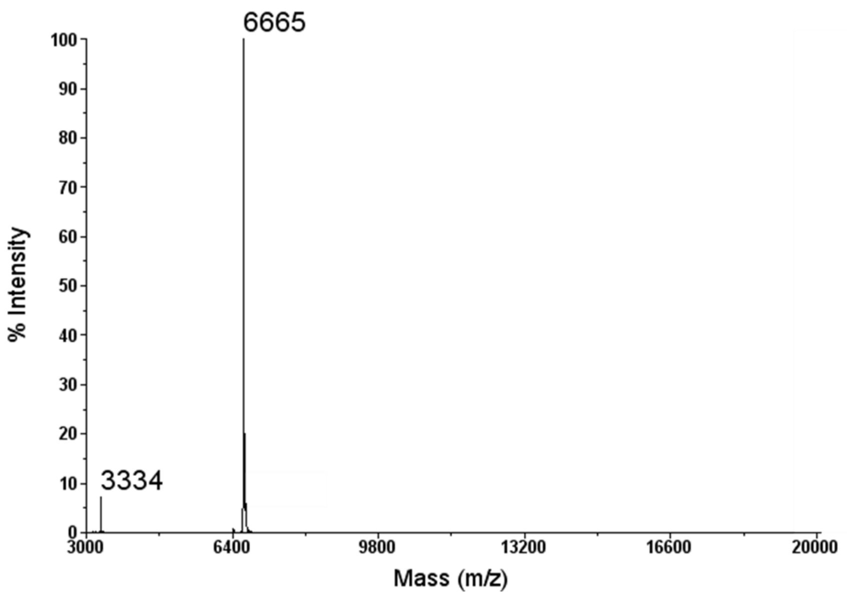

Figure S9. MALDI-TOF spectra with 3 to 20 kDa range after removal of free label. GB1 I6C/K28C sample labelled with MPSL overnight. Expected molecular weight: 6662 Da (doubly MPSL-labelled); 6425 Da (singly MPSL-labelled).

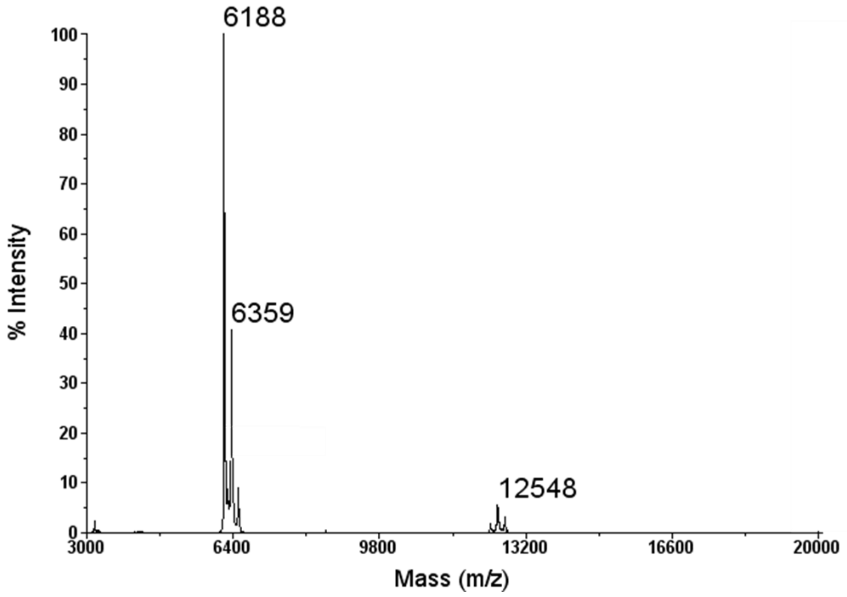

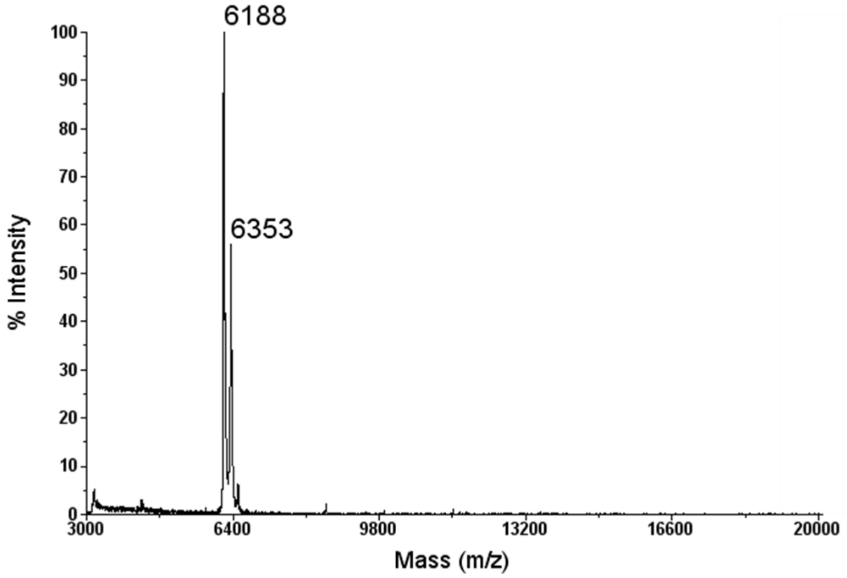

Figure S10. MALDI-TOF spectra with 3 to 20 kDa range after removal of free label. GB1 I6C/K28C sample labelled with IDSL at 3x (top) and 20x (bottom) IDSL-to-cysteine ratio overnight. Expected molecular weight: 6530 Da (doubly IDSL-labelled); 6359 Da (singly IDSL-labelled). Note the visible dimer mass for the 3x IDSL-to-cysteine ratio.

SDS-PAGE gel of spin-labelled GB1 6C/28C

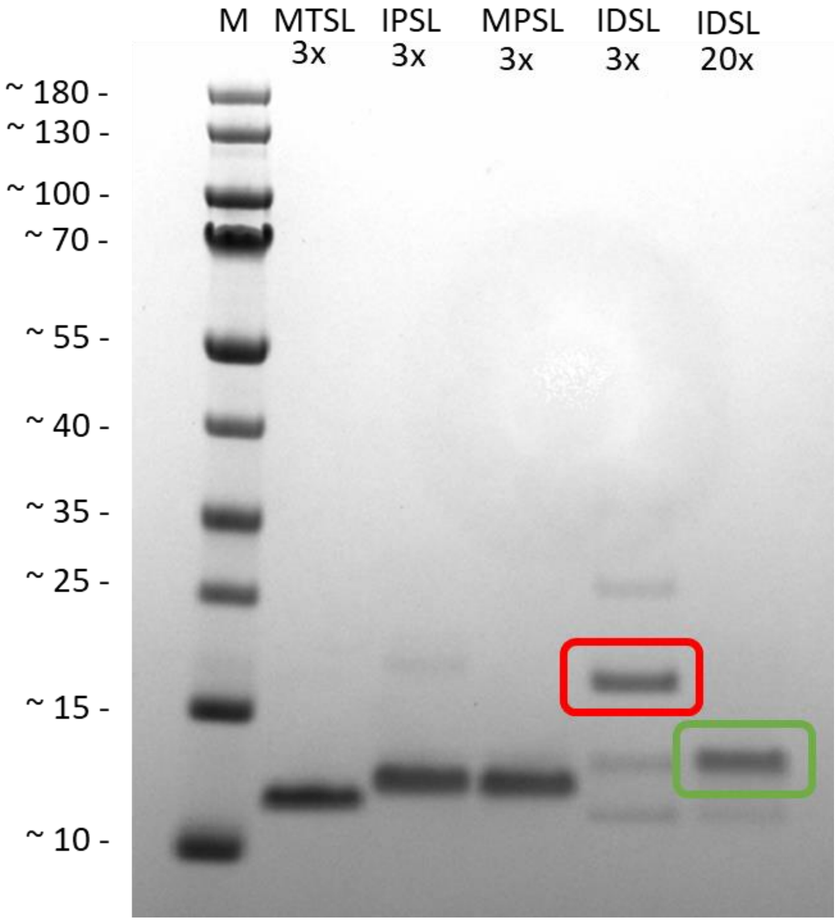

Figure S11. SDS-PAGE gel for spin-labelled GB1 6C/28C samples. Spin labels and label-to-cysteine ratios are indicated at the top of the gel. Samples were prepared without addition of reducing agent to the sample buffer to preserve the disulfide-linked dimer, clearly visible for the IDSL 3x sample (red circle). GB1 is known to run at a higher molecular weight on SDS gels, and the single strong bands observed for MTSL-, IPSL-, and MPSL-labelled GB1 suggest a single (fully labelled) moiety, while additional bands in the IDSL-labelled samples suggest presence of sub-moieties. The strongest band (green circle) for the IDSL 20x sample is also the largest size observed for this sample, confirming the CW results with >90% labelling efficiency.

Superposition of distance distributions of protonated and deuterated samples

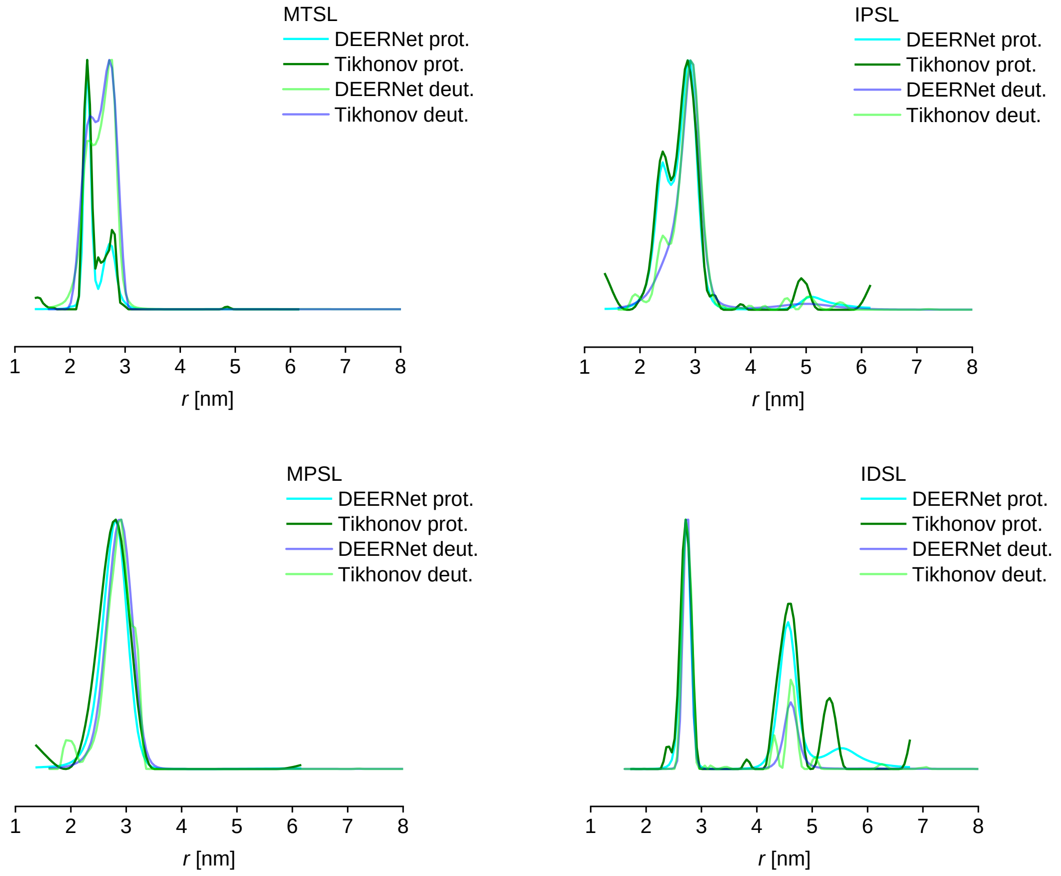

Figure S12. Superposition of distance distributions obtained for protonated and deuterated samples at 3:1 label-to-cysteine ratio and overnight labelling.

Refocused echo decay (Hahn echo) experiments

Refocused echo decay experiments illustrate the gain in distance resolution that can be obtained with deuteration of the samples. Shorter distances seem to correlate with closer proximity to protein protons that were not exchanged to deuterons and thus faster dephasing.

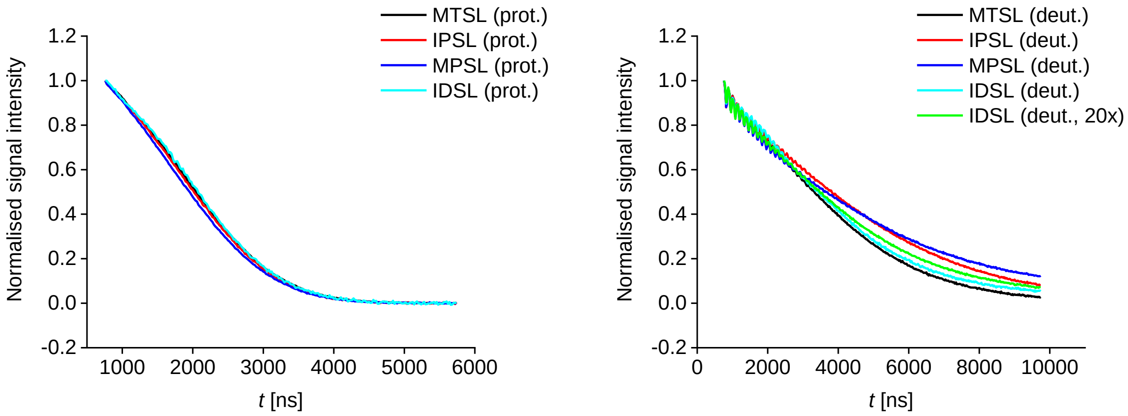

Figure S13. Refocused echo decay experiments. Left: protonated samples; right: deuterated samples.

Additional modelling

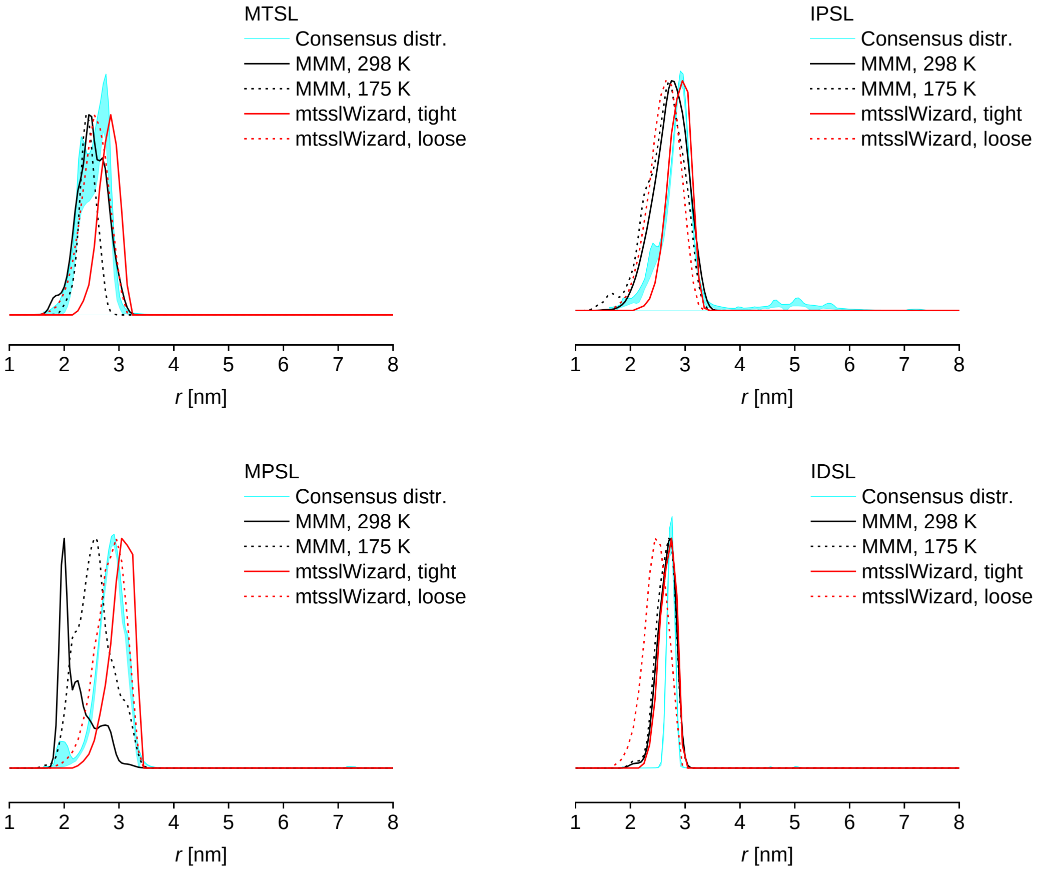

Figure S14. Comparison of different modelling approaches with experimental data for spin-labelled GB1 6C/28C. Uncertainty bands of the experimental consensus distributions as obtained from the CDA for each label are shown as cyan shade. MMM models for 298 K (solid line) and 175 K (dashed line) are shown in black; mtsslWizard models for tight (solid line) and loose (dashed line) settings are shown in red.

CDA reports

In the following the full CDA reports are attached.

**Sample: spin-labelled GB1 6C/28C**	**Code**
MTSL deuterated (3:1 ratio)	211115_BEBQ56.24_DEER
IDSL deuterated (3:1 ratio)	200723_KAq142.3_yPELDOR
IDSL deuterated (20:1 ratio)	200729_KAq142.15_yPELDOR
MPSL deuterated (3:1 ratio)	200729_KAq142.9_yPELDOR
IPSL deuterated (3:1 ratio)	200724_KAq142.6_yPELDOR
MTSL protonated (3:1 ratio)	200302_KAq137.4_yPELDOR_2.4us
IDSL protonated (3:1 ratio)	200223_BEBQ40.11_PELDOR
MPSL protonated (3:1 ratio)	200223_BEBQ40.4_PELDOR
IPSL protonated (3:1 ratio)	200223_BEBQ40.16_PELDOR

DEER analysis report on dataset 211115_BEBQ56.24_DEER

DEERNet Spinach SVN Rev 5501 and DeerLab 0.9.1 Tikhonov regularization

ConsensusDEERAnalyzer

see: S. G. Worswick et al., DOI: 10.1126/sciadv.aat5218, L. Fabregas Ibanez et al., DOI: 10.5194/mr-1-209-2020

15-Nov-2021 09:46:14

1. Distance distributions

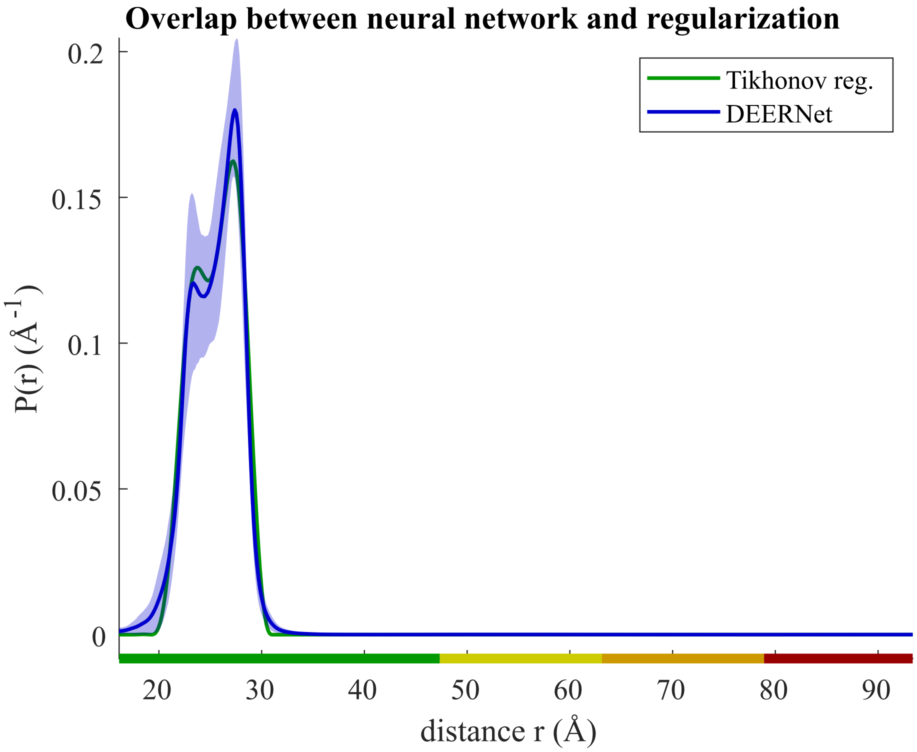

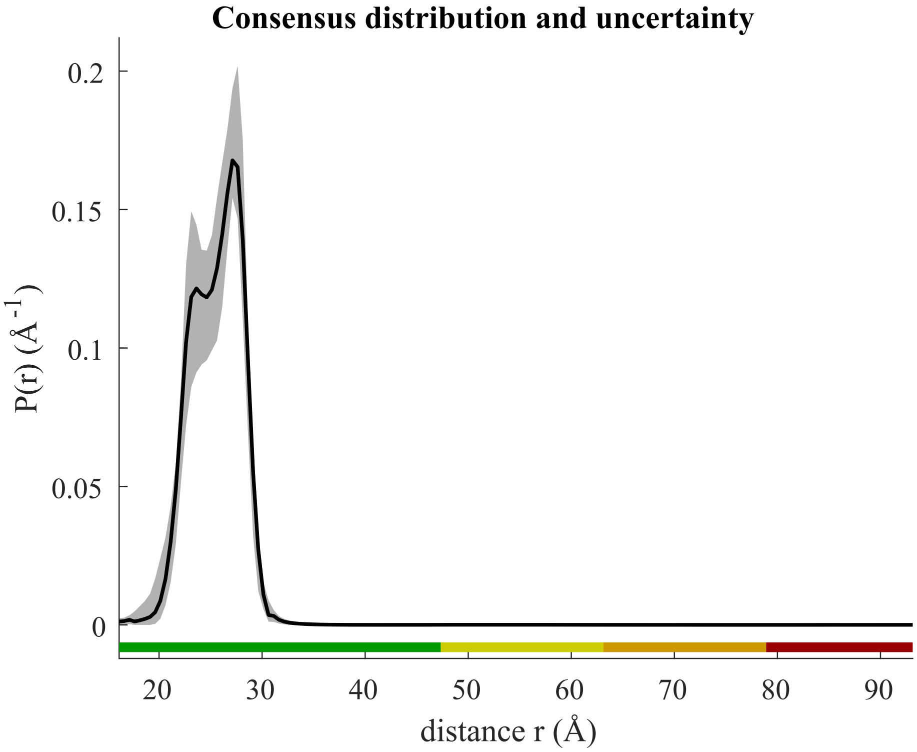

2. Fits of time-domain data

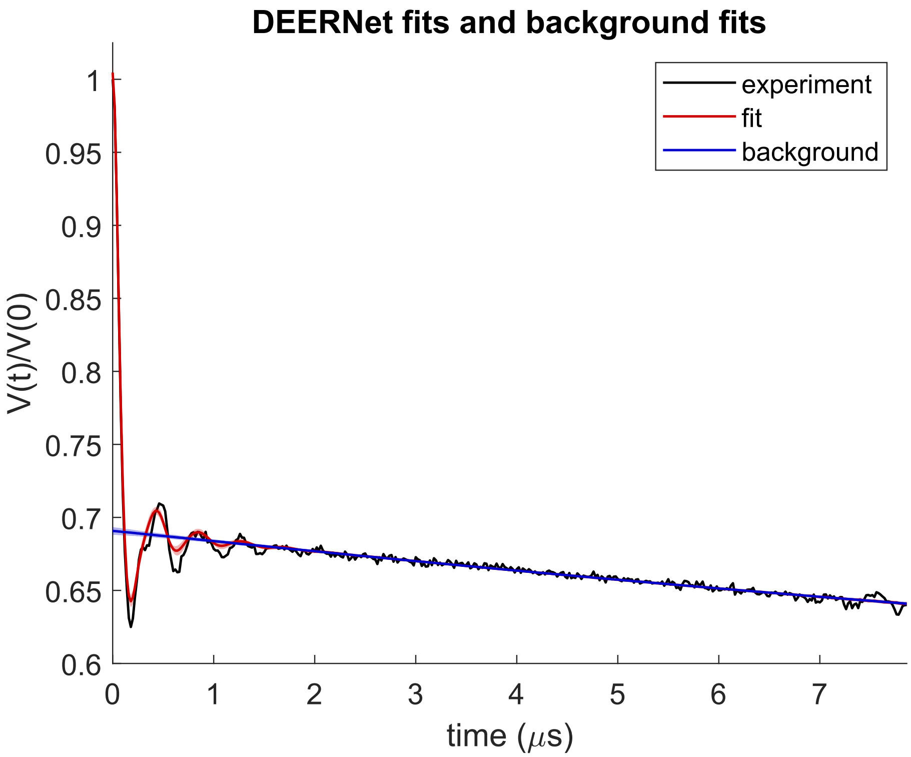

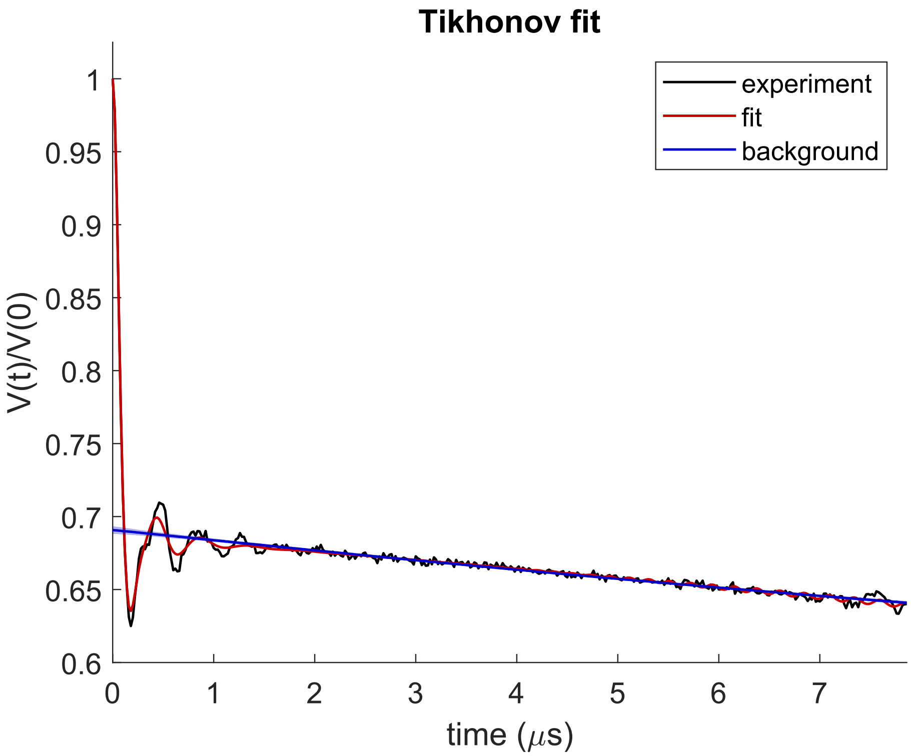

3. Experimental and processing parameters

Time increment: 20 ns

Maximum time: 7860 ns

Zero time: 296 ns

Phase: 12.3 degree

Noise estimates normalized to maximum signal

From imaginary part: 0.00190

From DEERNet fit: 0.00359

From Tikhonov fit: 0.00320

Modulation depth: 0.309

Signal-to-noise ratio: 86.2 (w.r.t. modulation)

Ensemble of 24 neural networks

Background separation by neural network

Regularization parameter by best overlap with neural network solution

Regularization parameter used: 1.47

Reg. par. initial estimate by GCV: 0.09

Overlap between DEERNet and regularization solutions: 0.950

Mean distance: 25.6 Å

Distance standard deviation: 2.9 Å

Full data set in Matlab format: C:\Users\Bela\OneDrive - University of St Andrews\Spectra\BEBQ56\211115_BEBQ56.24_DEER_consensus_DEER_analysis.mat

Distance distributions in text format: C:\Users\Bela\OneDrive - University of St Andrews\Spectra\BEBQ56\211115_BEBQ56.24_DEER_consensus_DEER_distribution.dat

Fit and background in text format: C:\Users\Bela\OneDrive - University of St Andrews\Spectra\BEBQ56\211115_BEBQ56.24_DEER_consensus_DEER_fit.dat

DEER analysis report on dataset 200723_KAq142.3_yPELDOR

DEERNet Spinach SVN Rev 5662 and DeerLab 0.9.1 Tikhonov regularization

ComparativeDEERAnalyzer

see: S. G. Worswick et al., DOI: 10.1126/sciadv.aat5218, L. Fabregas Ibanez et al., DOI: 10.5194/mr-1-209-2020

25-Oct-2021 11:43:40

1. Distance distributions

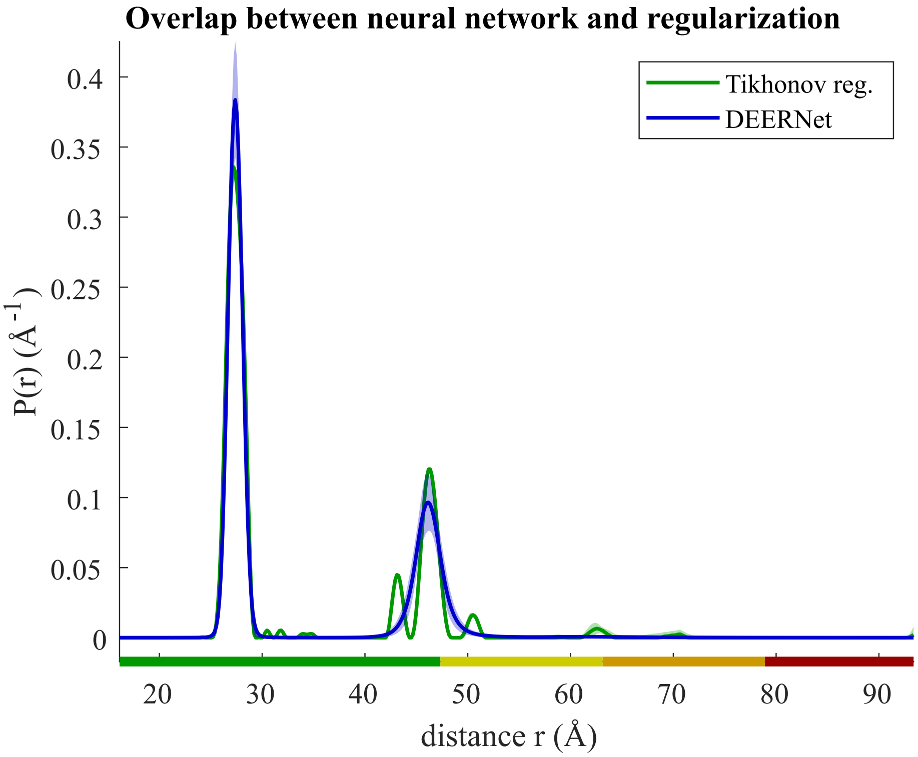

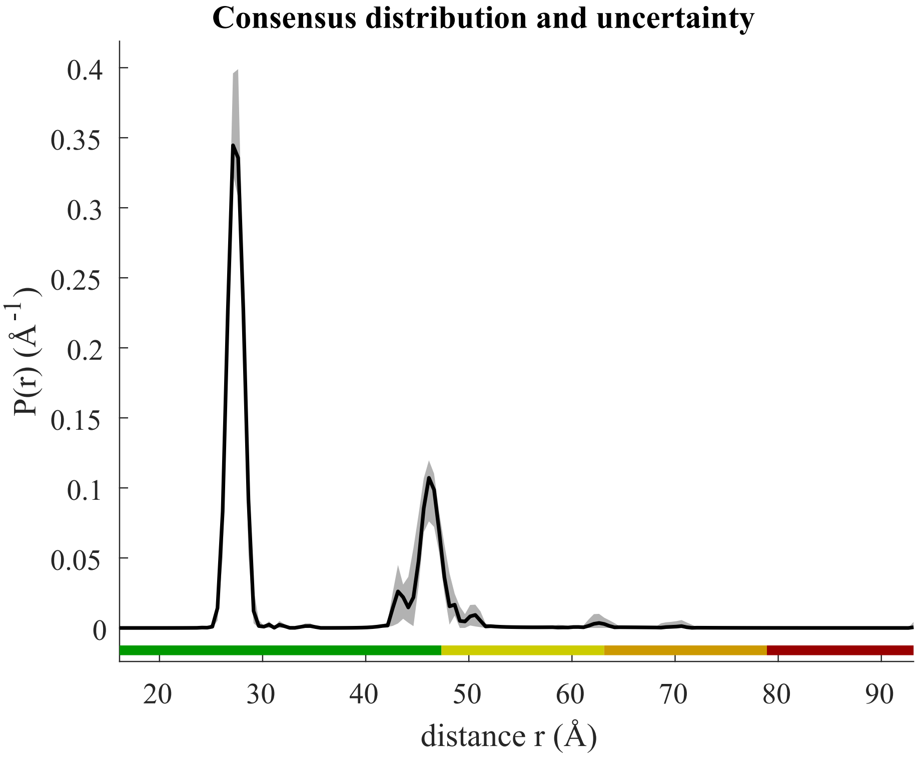

2. Fits of time-domain data

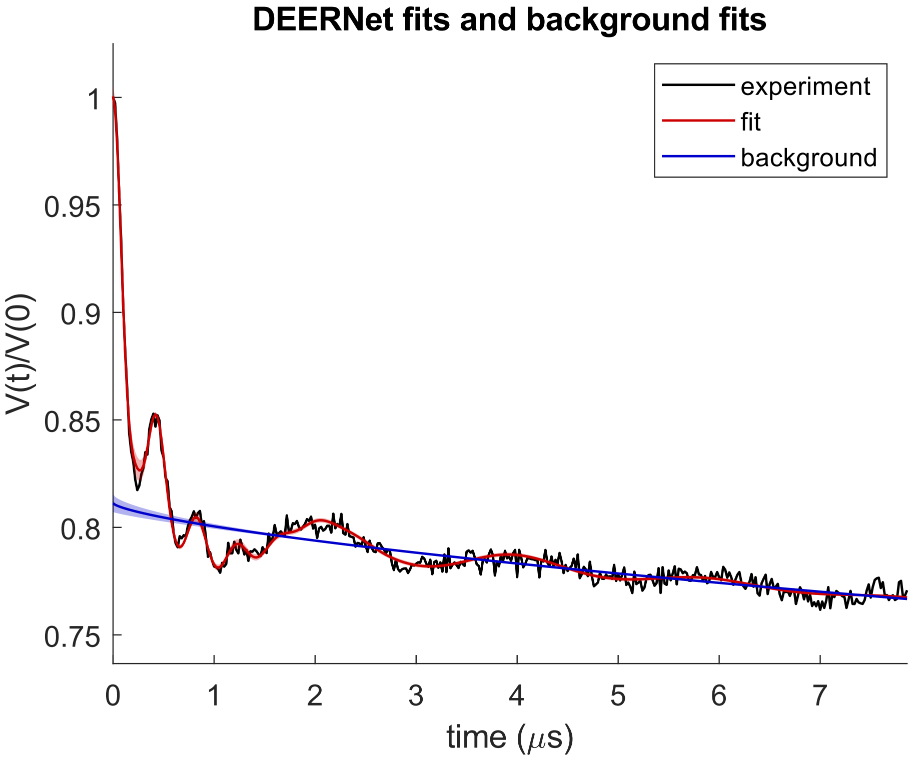

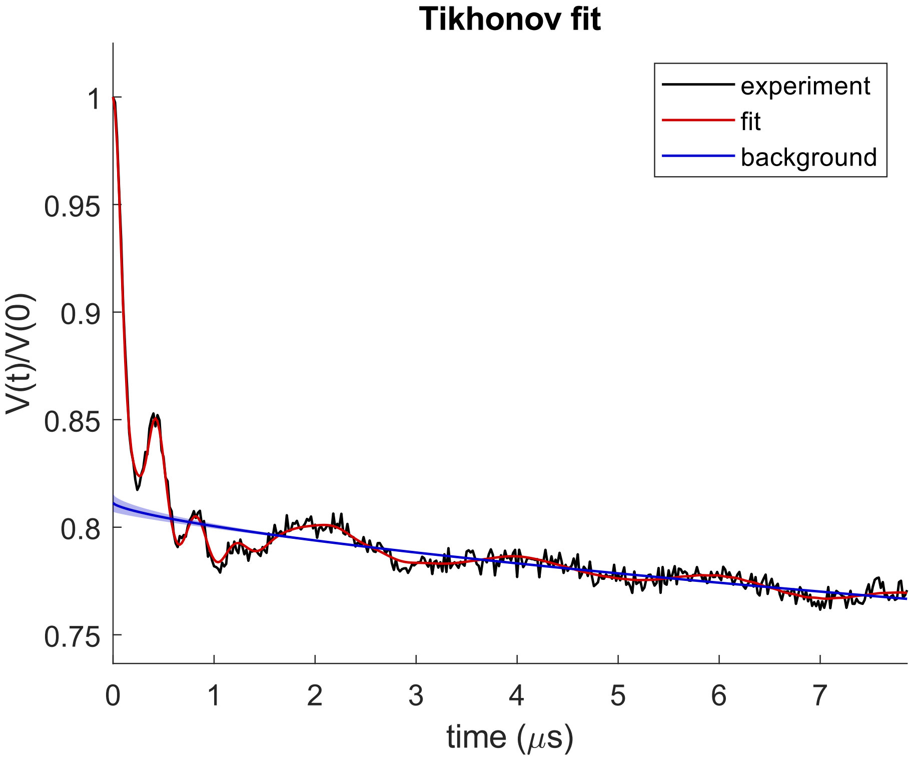

3. Experimental and processing parameters

Time increment: 20 ns

Maximum time: 7860 ns

Zero time: 295 ns

Phase: -2.0 degree

Noise estimates normalized to maximum signal

From imaginary part: 0.00276

From DEERNet fit: 0.00330

From Tikhonov fit: 0.00308

Modulation depth: 0.189

Signal-to-noise ratio: 57.1 (w.r.t. modulation)

Ensemble of 32 neural networks

Background separation by neural network

Regularization parameter by best overlap with neural network solution

Regularization parameter used: 0.14

Reg. par. initial estimate by L-curve corner: 1.58

Overlap between DEERNet and regularization solutions: 0.856

Mean distance: 18.8 Å

Distance standard deviation: 15.4 Å

Full data set in Matlab format: 200723_KAq142.3_yPELDOR_consensus_DEER_analysis.mat

Distance distributions in text format:
200723_KAq142.3_yPELDOR_consensus_DEER_distribution.dat

Fit and background in text format: 200723_KAq142.3_yPELDOR_consensus_DEER_fit.dat

DEER analysis report on dataset 200729_KAq142.15_yPELDOR

DEERNet Spinach SVN Rev 5662 and DeerLab 0.9.1 Tikhonov regularization

ComparativeDEERAnalyzer

see: S. G. Worswick et al., DOI: 10.1126/sciadv.aat5218, L. Fabregas Ibanez et al., DOI: 10.5194/mr-1-209-2020

25-Oct-2021 11:46:48

1. Distance distributions

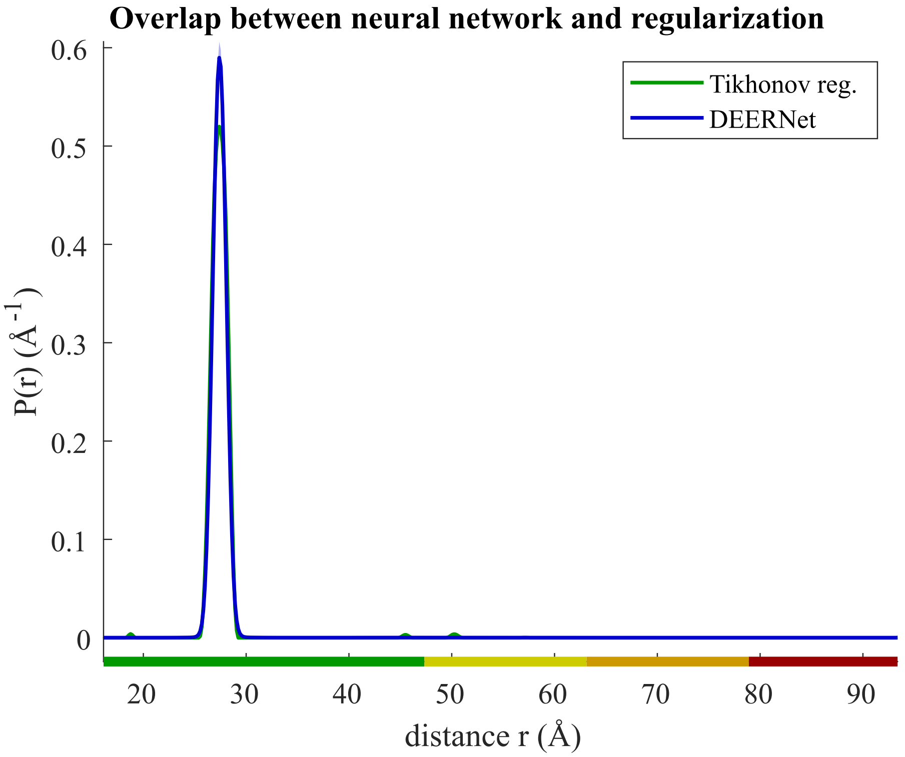

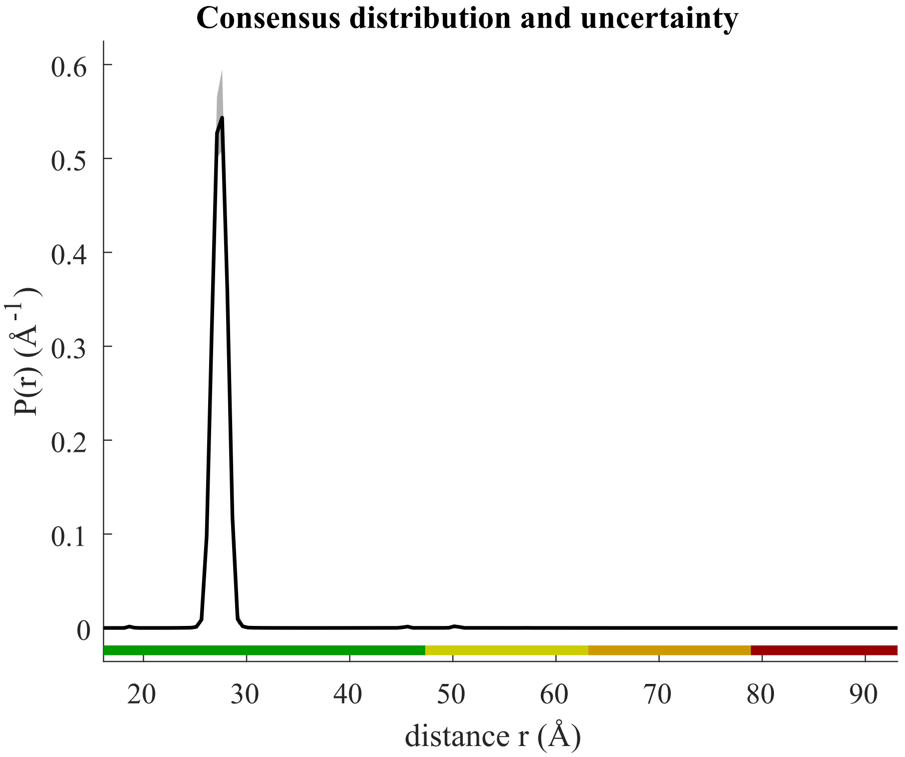

2. Fits of time-domain data

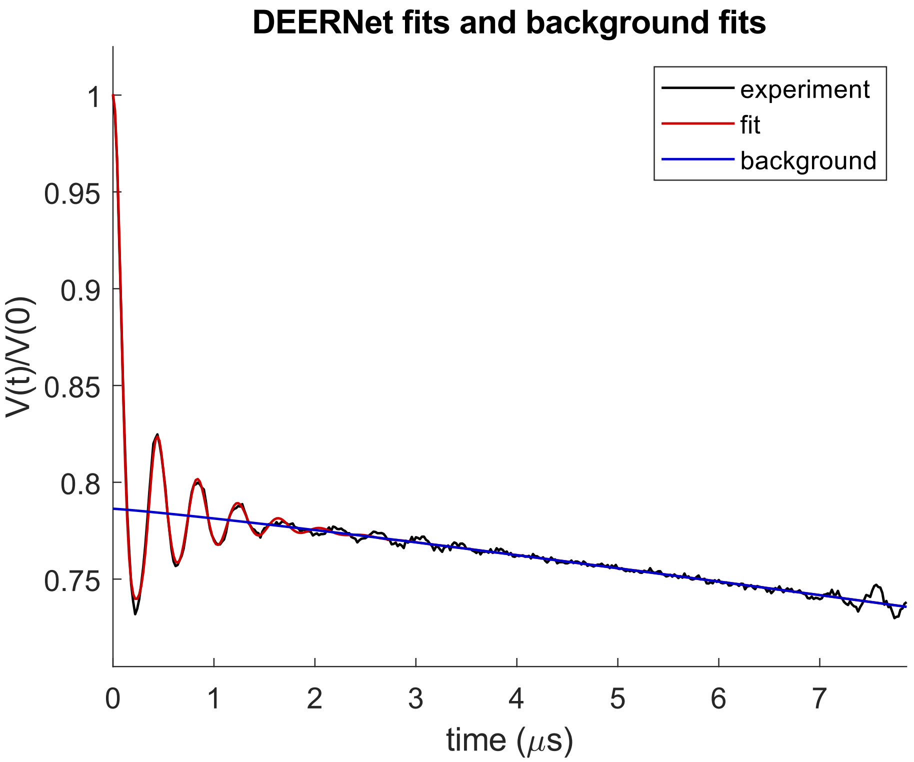

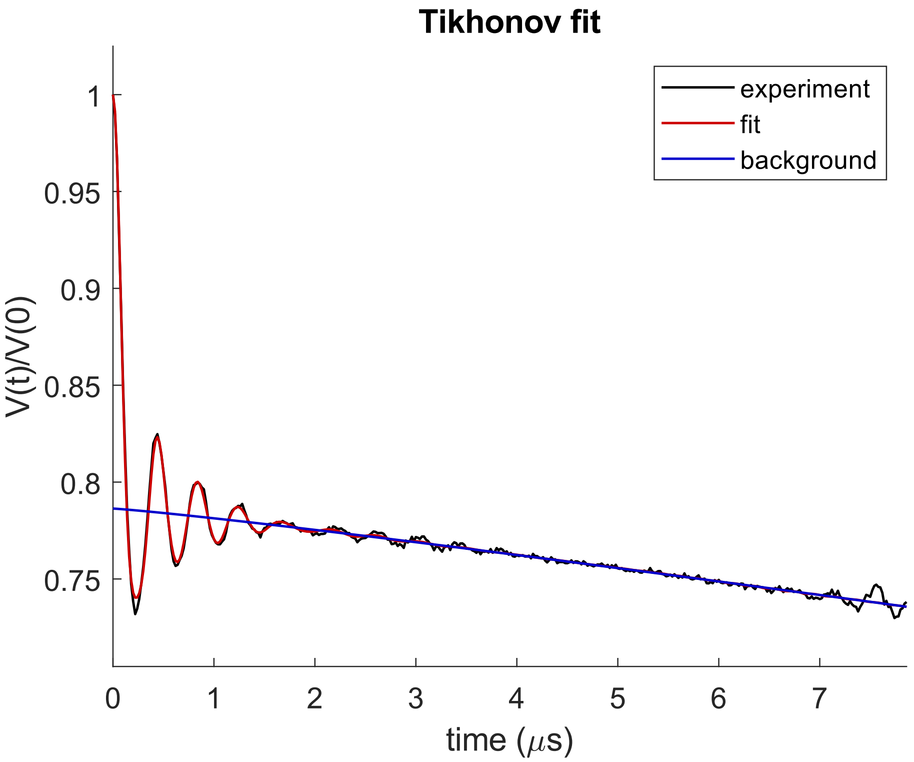

3. Experimental and processing parameters

Time increment: 20 ns

Maximum time: 7860 ns

Zero time: 296 ns

Phase: -1.8 degree

Noise estimates normalized to maximum signal

From imaginary part: 0.00125

From DEERNet fit: 0.00206

From Tikhonov fit: 0.00194

Modulation depth: 0.214

Signal-to-noise ratio: 103.5 (w.r.t. modulation)

Ensemble of 32 neural networks

Background separation by neural network

Regularization parameter by best overlap with neural network solution

Regularization parameter used: 0.10

Reg. par. initial estimate by GCV: 0.00

Overlap between DEERNet and regularization solutions: 0.934

Mean distance: 27.4 Å

Distance standard deviation: 0.6 Å

Full data set in Matlab format: 200729_KAq142.15_yPELDOR_consensus_DEER_analysis.mat

Distance distributions in text format: 200729_KAq142.15_yPELDOR_consensus_DEER_distribution.dat

Fit and background in text format: 200729_KAq142.15_yPELDOR_consensus_DEER_fit.dat

DEER analysis report on dataset 200729_KAq142.9_yPELDOR

DEERNet Spinach SVN Rev 5662 and DeerLab 0.9.1 Tikhonov regularization

ComparativeDEERAnalyzer

see: S. G. Worswick et al., DOI: 10.1126/sciadv.aat5218, L. Fabregas Ibanez et al., DOI: 10.5194/mr-1-209-2020

25-Oct-2021 11:45:59

1. Distance distributions

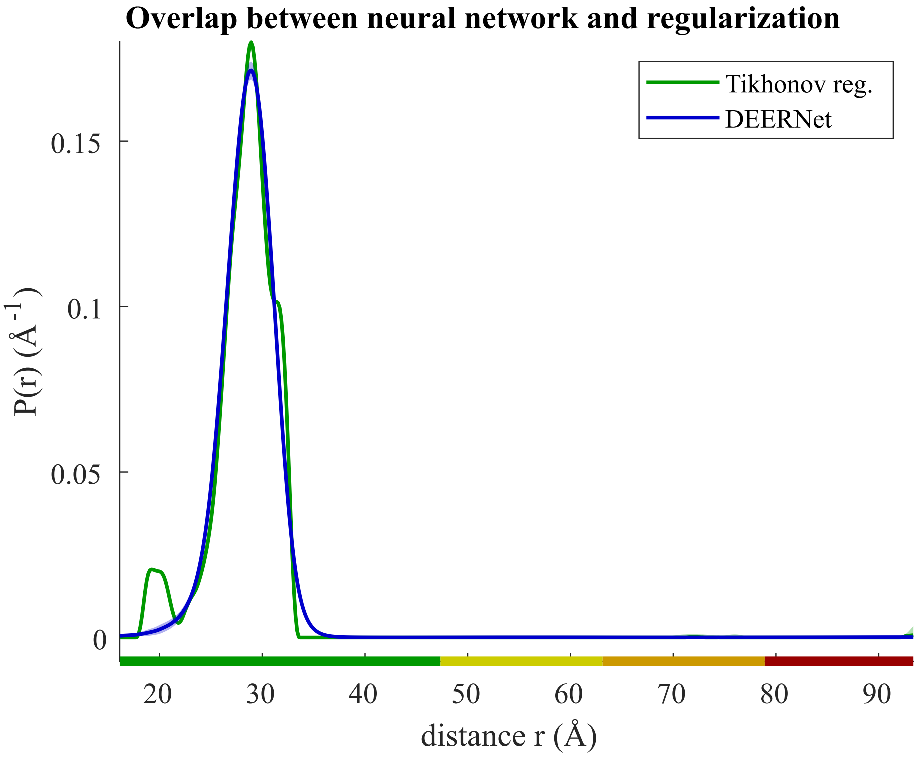

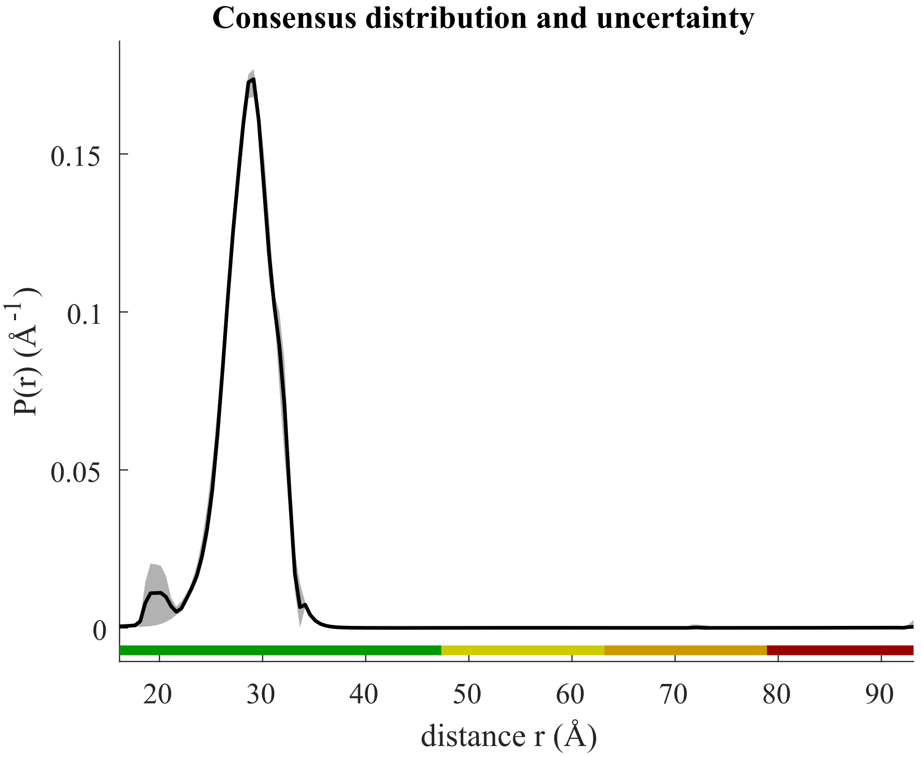

2. Fits of time-domain data

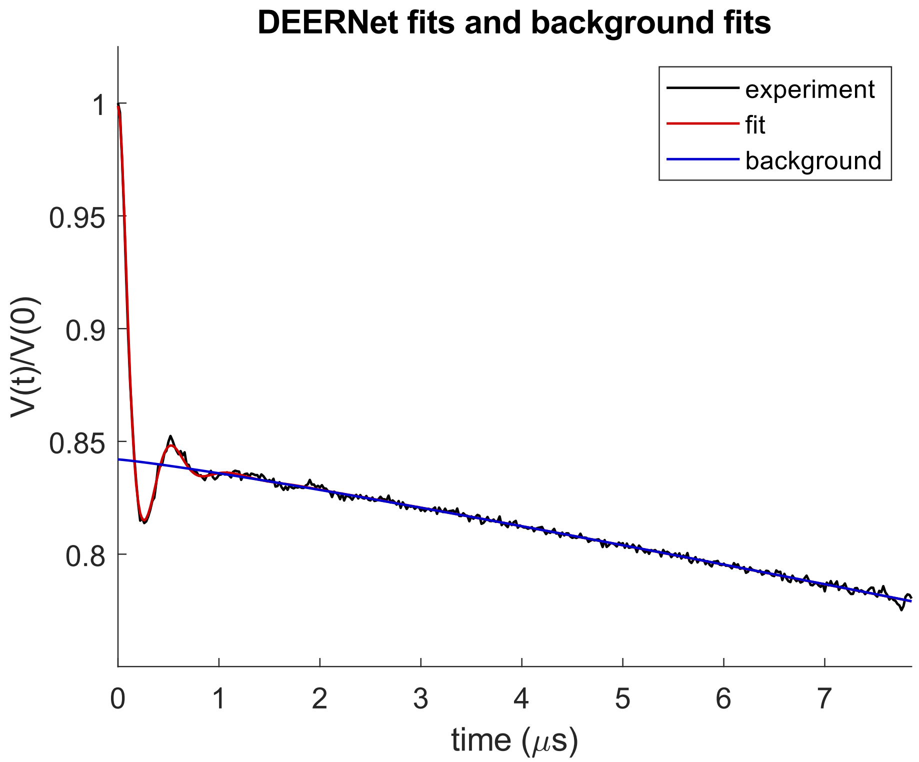

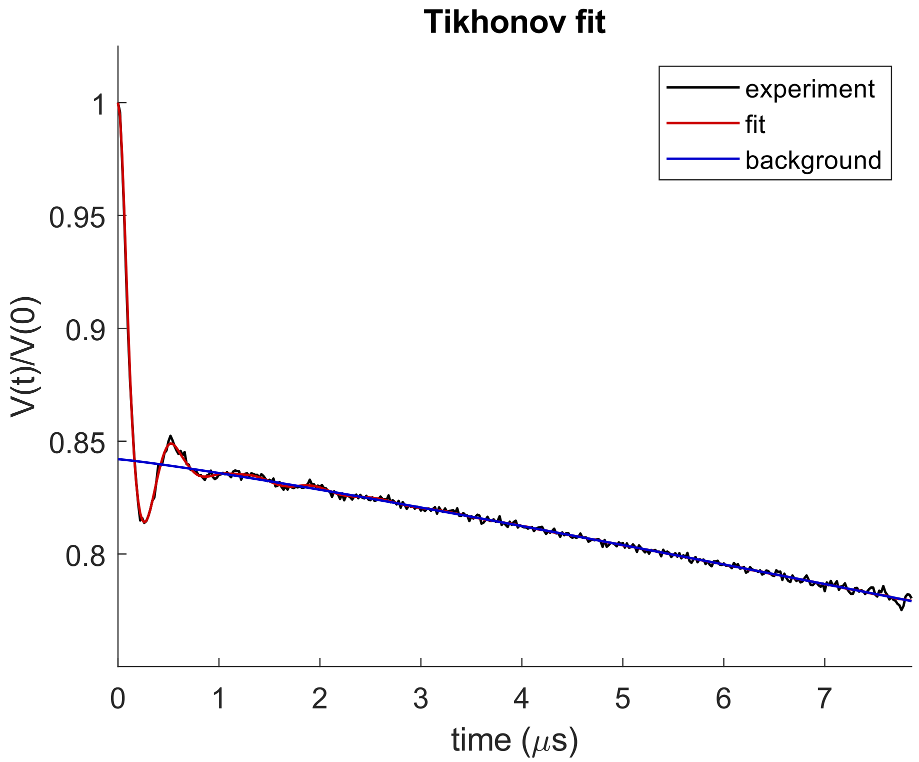

3. Experimental and processing parameters

Time increment: 20 ns

Maximum time: 7860 ns

Zero time: 296 ns

Phase: -6.3 degree

Noise estimates normalized to maximum signal

From imaginary part: 0.00479

From DEERNet fit: 0.00133

From Tikhonov fit: 0.00123

Modulation depth: 0.158

Signal-to-noise ratio: 119.0 (w.r.t. modulation)

Ensemble of 32 neural networks

Background separation by neural network

Regularization parameter by best overlap with neural network solution

Regularization parameter used: 0.25

Reg. par. initial estimate by GCV: 0.25

Overlap between DEERNet and regularization solutions: 0.920

Mean distance: 28.5 Å

Distance standard deviation: 2.0 Å

Full data set in Matlab format: 200729_KAq142.9_yPELDOR_consensus_DEER_analysis.mat

Distance distributions in text format:
200729_KAq142.9_yPELDOR_consensus_DEER_distribution.dat

Fit and background in text format: 200729_KAq142.9_yPELDOR_consensus_DEER_fit.dat

DEER analysis report on dataset 200724_KAq142.6_yPELDOR

DEERNet Spinach SVN Rev 5662 and DeerLab 0.9.1 Tikhonov regularization

ComparativeDEERAnalyzer

see: S. G. Worswick et al., DOI: 10.1126/sciadv.aat5218, L. Fabregas Ibanez et al., DOI: 10.5194/mr-1-209-2020

25-Oct-2021 11:45:05

1. Distance distributions

Overlap between neural network and regularization

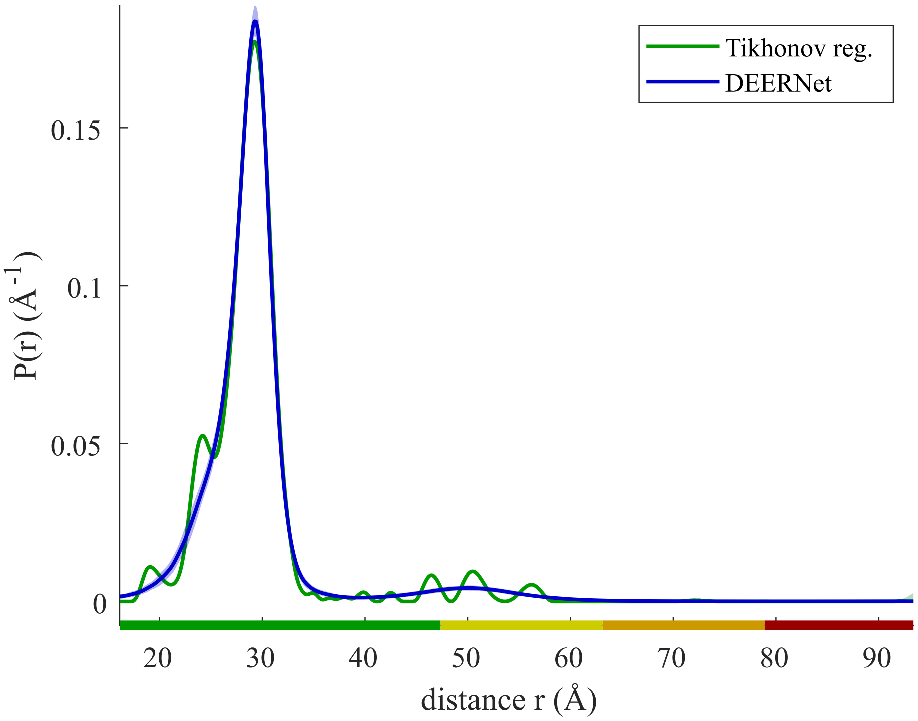

Consensus distribution and uncertainty

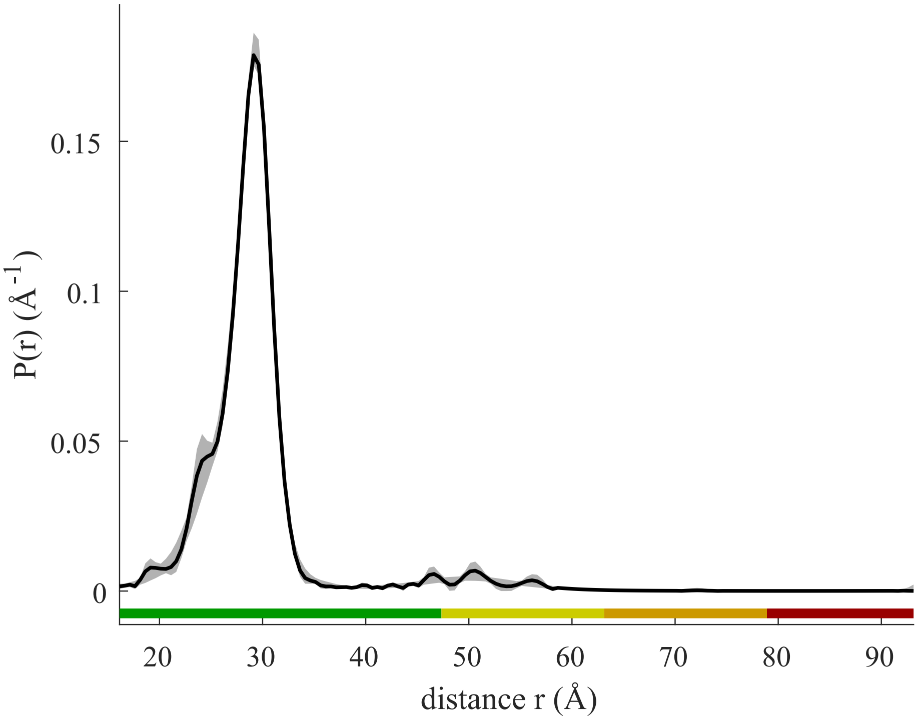

2. Fits of time-domain data

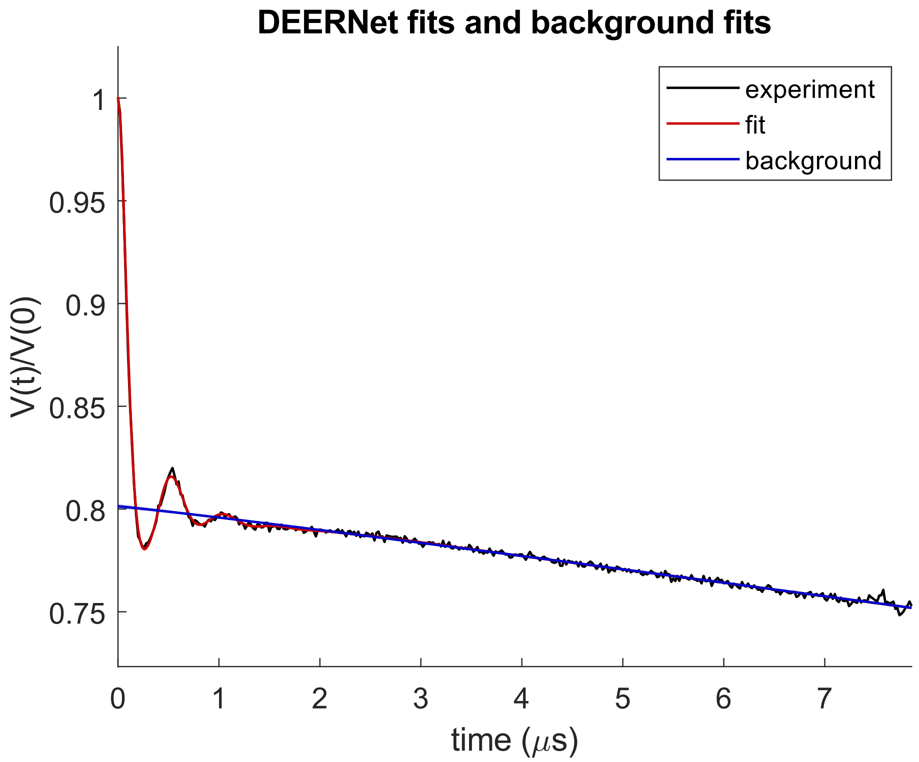

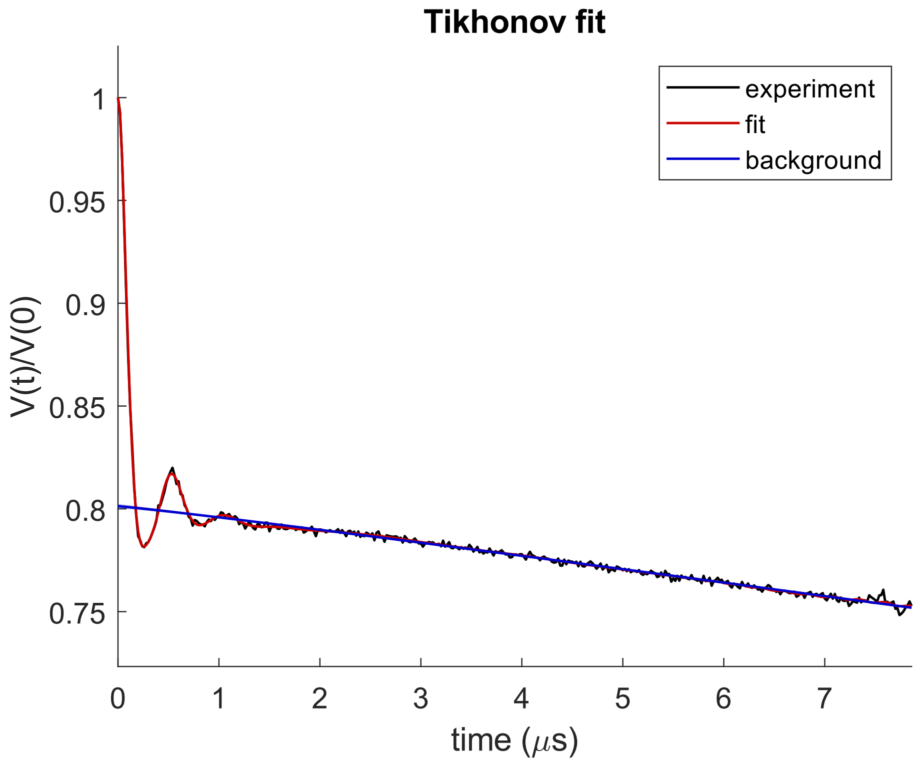

3. Experimental and processing parameters

Time increment: 20 ns

Maximum time: 7860 ns

Zero time: 296 ns

Phase: -4.8 degree

Noise estimates normalized to maximum signal

From imaginary part: 0.00114

From DEERNet fit: 0.00131

From Tikhonov fit: 0.00124

Modulation depth: 0.199

Signal-to-noise ratio: 151.7 (w.r.t. modulation)

Ensemble of 32 neural networks

Background separation by neural network

Regularization parameter by best overlap with neural network solution

Regularization parameter used: 0.44

Reg. par. initial estimate by GCV: 0.44

Overlap between DEERNet and regularization solutions: 0.932

Mean distance: 28.5 Å

Distance standard deviation: 2.3 Å

Full data set in Matlab format: 200724_KAq142.6_yPELDOR_consensus_DEER_analysis.mat

Distance distributions in text format: 200724_KAq142.6_yPELDOR_consensus_DEER_distribution.dat

Fit and background in text format: 200724_KAq142.6_yPELDOR_consensus_DEER_fit.dat

DEER analysis report on dataset 200302_KAq137.4_yPELDOR_2.4us

DEERNet Spinach SVN Rev 5662 and DeerLab 0.9.1 Tikhonov regularization

ComparativeDEERAnalyzer

see: S. G. Worswick et al., DOI: 10.1126/sciadv.aat5218, L. Fabregas Ibanez et al., DOI: 10.5194/mr-1-209-2020

25-Oct-2021 11:47:12

1. Distance distributions

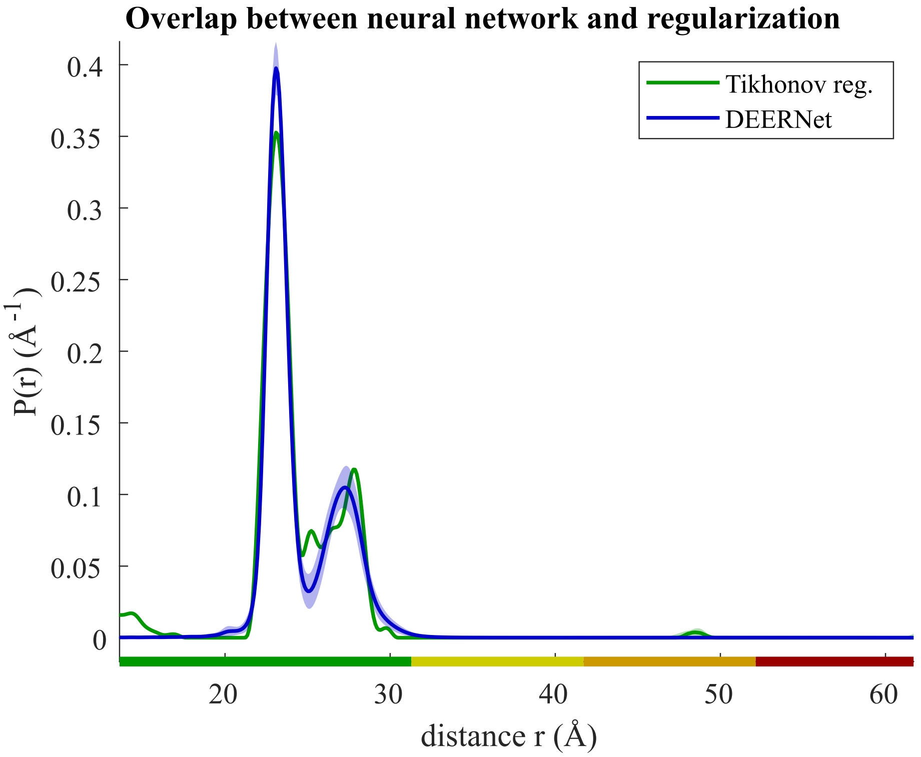

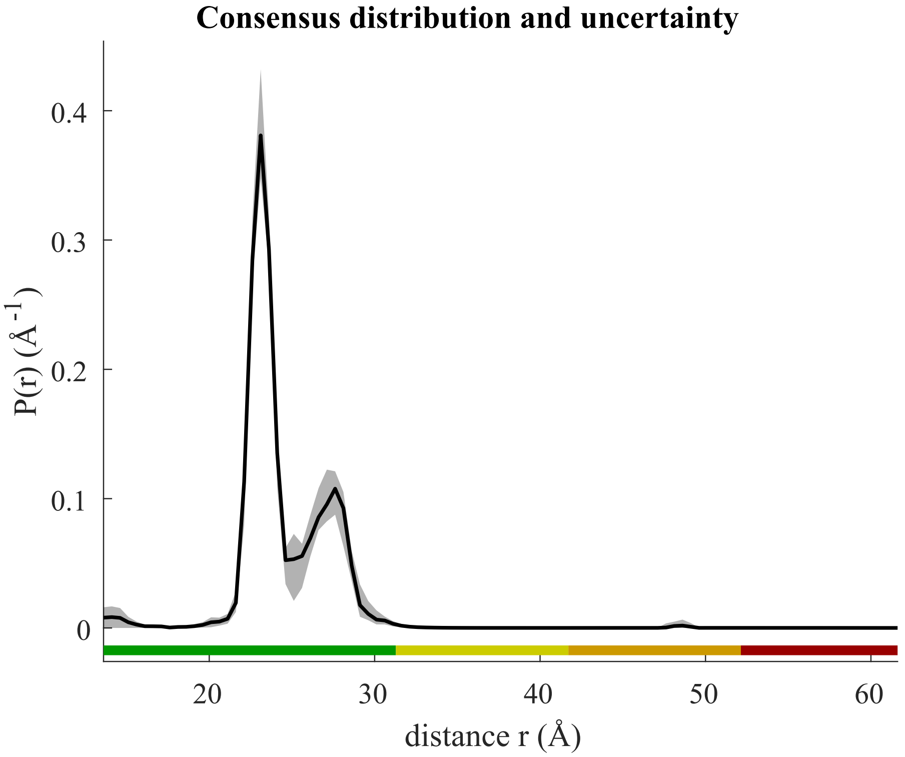

2. Fits of time-domain data

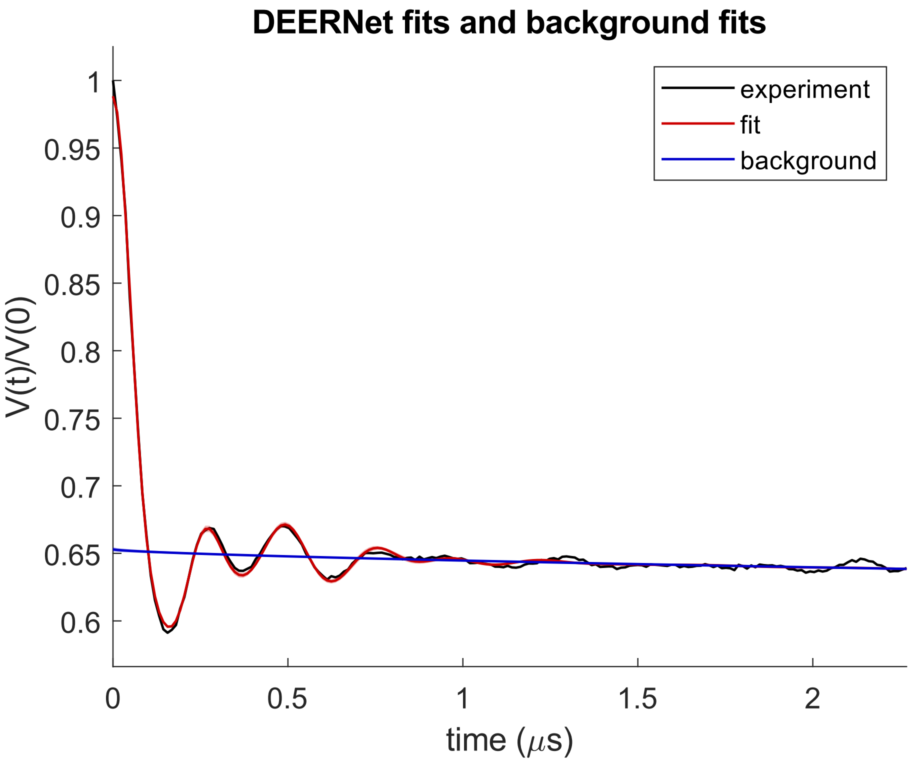

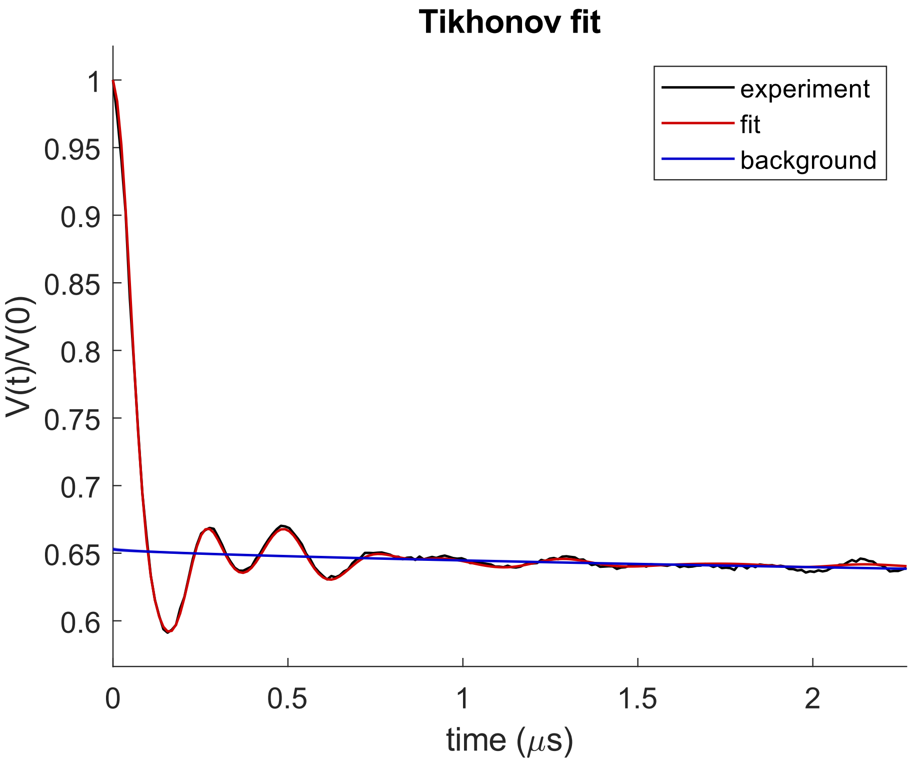

3. Experimental and processing parameters

Time increment: 12 ns

Maximum time: 2.268000e+03 ns

Zero time: 115 ns

Phase: -3.1 degree

Noise estimates normalized to maximum signal

From imaginary part: 0.00156

From DEERNet fit: 0.00247

From Tikhonov fit: 0.00220

Modulation depth: 0.347

Signal-to-noise ratio: 140.4 (w.r.t. modulation)

Ensemble of 32 neural networks

Background separation by neural network

Regularization parameter by best overlap with neural network solution

Regularization parameter used: 0.16

Reg. par. initial estimate by L-curve corner: 0.32

Overlap between DEERNet and regularization solutions: 0.888

Mean distance: 24.1 Å

Distance standard deviation: 2.3 Å

Full data set in Matlab format:
200302_KAq137.4_yPELDOR_2.4us_consensus_DEER_analysis.mat

Distance distributions in text format:
200302_KAq137.4_yPELDOR_2.4us_consensus_DEER_distribution.dat

Fit and background in text format:
200302_KAq137.4_yPELDOR_2.4us_consensus_DEER_fit.dat

DEER analysis report on dataset 200223_BEBQ40.11_PELDOR

DEERNet Spinach SVN Rev 5662 and DeerLab 0.9.1 Tikhonov regularization

ComparativeDEERAnalyzer

see: S. G. Worswick et al., DOI: 10.1126/sciadv.aat5218, L. Fabregas Ibanez et al., DOI: 10.5194/mr-1-209-2020

25-Oct-2021 11:49:02

1. Distance distributions

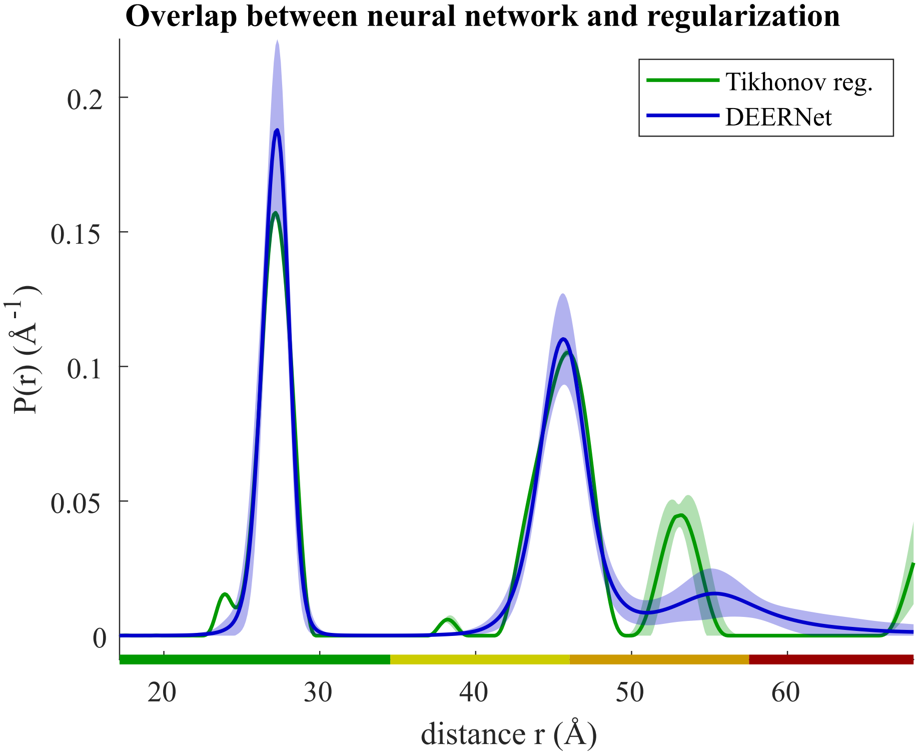

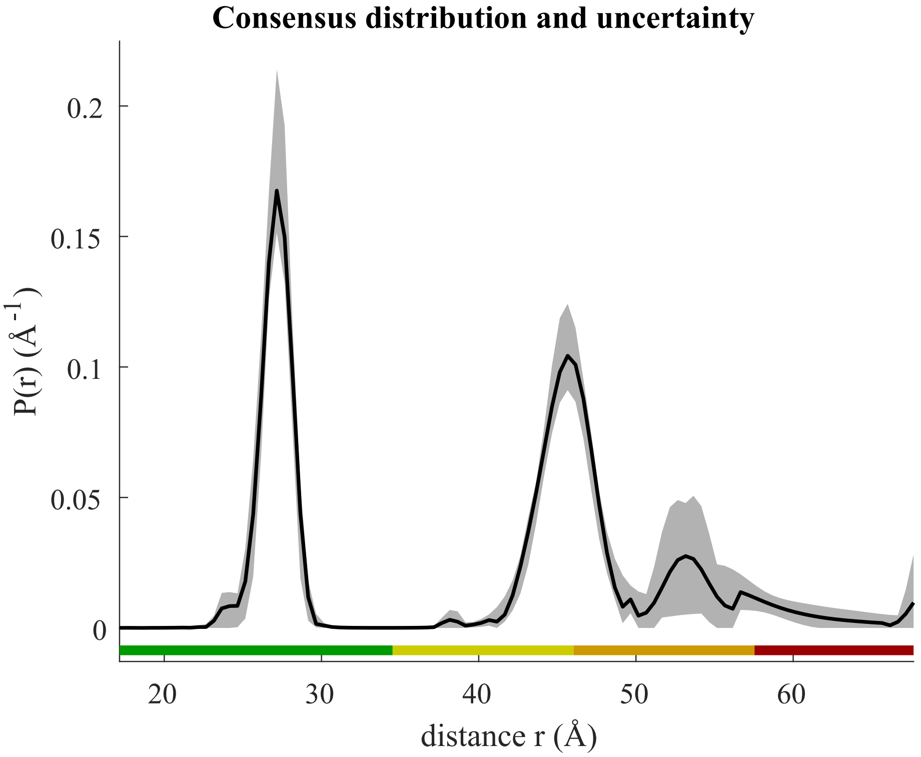

2. Fits of time-domain data

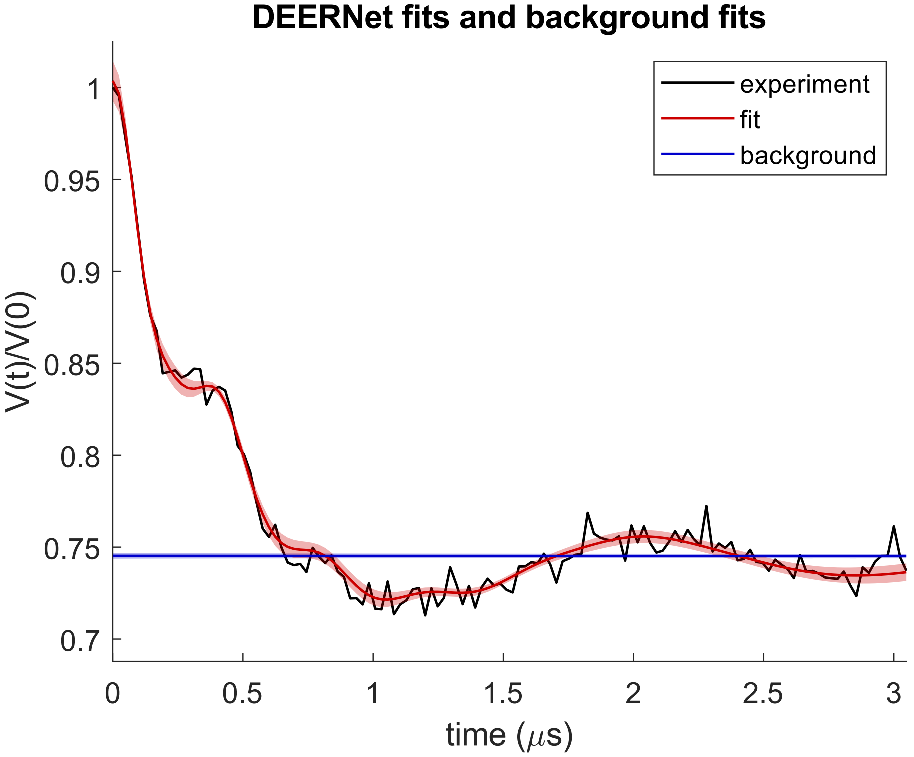

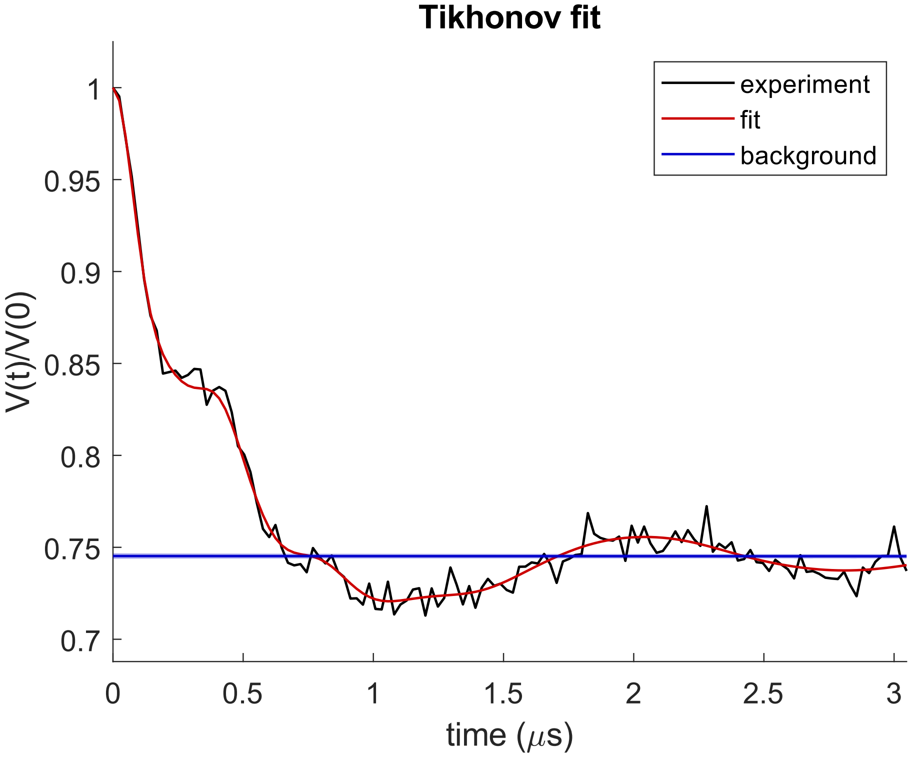

3. Experimental and processing parameters

Time increment: 24 ns

Maximum time: 3048 ns

Zero time: 109 ns

Phase: -3.5 degree

Noise estimates normalized to maximum signal

From imaginary part: 0.00665

From DEERNet fit: 0.00652

From Tikhonov fit: 0.00623

Modulation depth: 0.255

Signal-to-noise ratio: 39.1 (w.r.t. modulation)

Ensemble of 32 neural networks

Background separation by neural network

Regularization parameter by best overlap with neural network solution

Regularization parameter used: 0.56

Reg. par. initial estimate by L-curve corner: 6.31

Overlap between DEERNet and regularization solutions: 0.840

Mean distance: 89.5 Å

Distance standard deviation: 32.1 Å

Full data set in Matlab format: 200223_BEBQ40.11_PELDOR_consensus_DEER_analysis.mat

Distance distributions in text format: 200223_BEBQ40.11_PELDOR_consensus_DEER_distribution.dat

Fit and background in text format: 200223_BEBQ40.11_PELDOR_consensus_DEER_fit.dat

DEER analysis report on dataset 200223_BEBQ40.4_PELDOR

DEERNet Spinach SVN Rev 5662 and DeerLab 0.9.1 Tikhonov regularization

ComparativeDEERAnalyzer

see: S. G. Worswick et al., DOI: 10.1126/sciadv.aat5218, L. Fabregas Ibanez et al., DOI: 10.5194/mr-1-209-2020

25-Oct-2021 11:47:37

1. Distance distributions

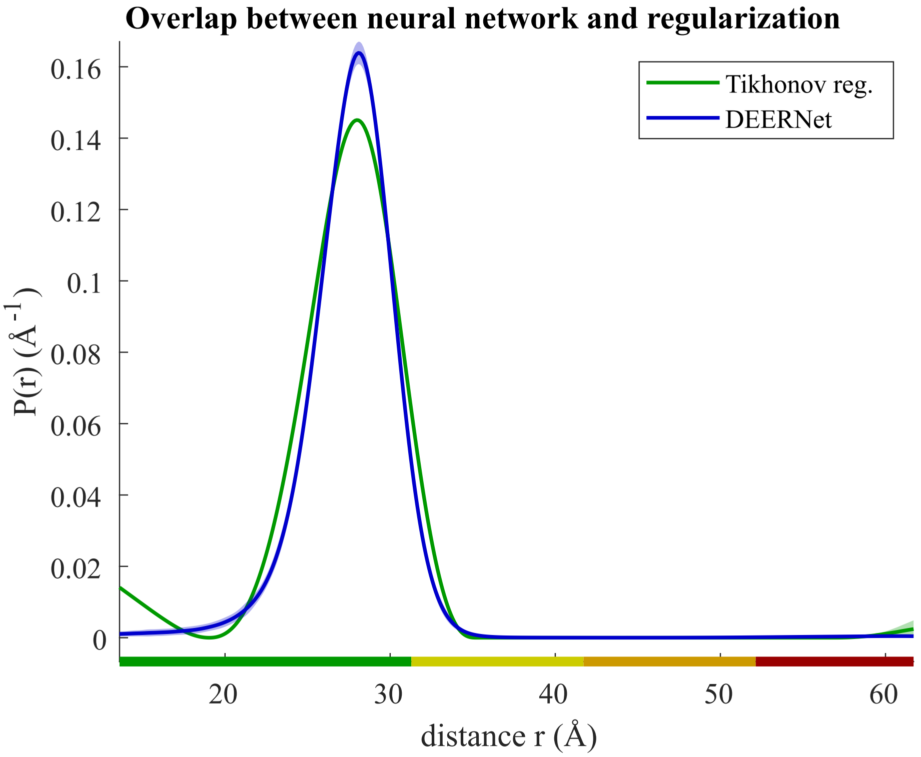

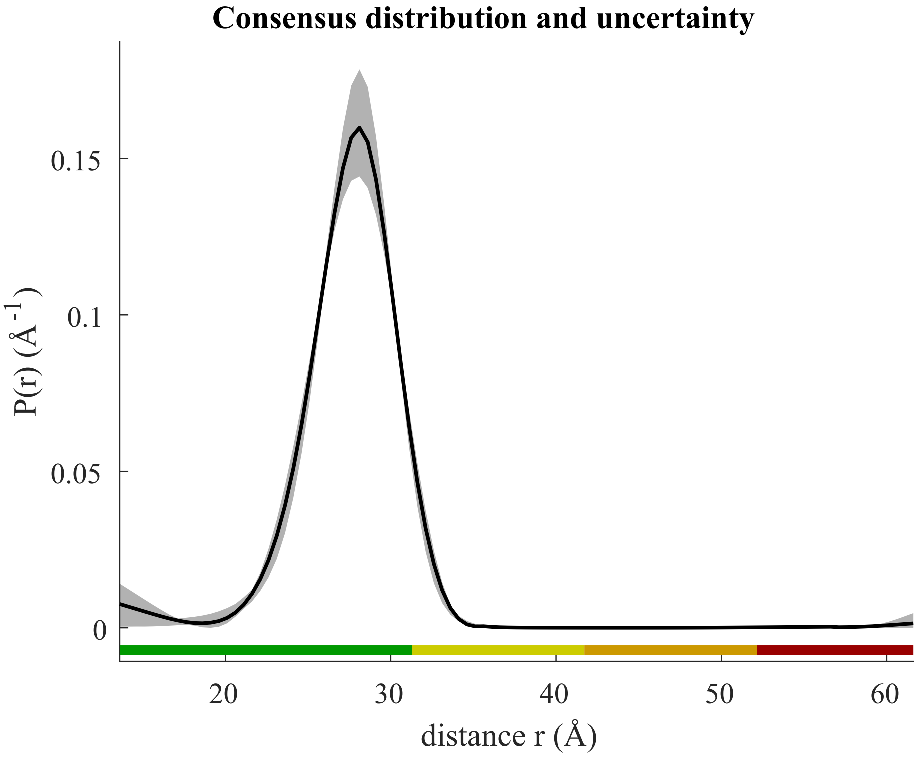

2. Fits of time-domain data

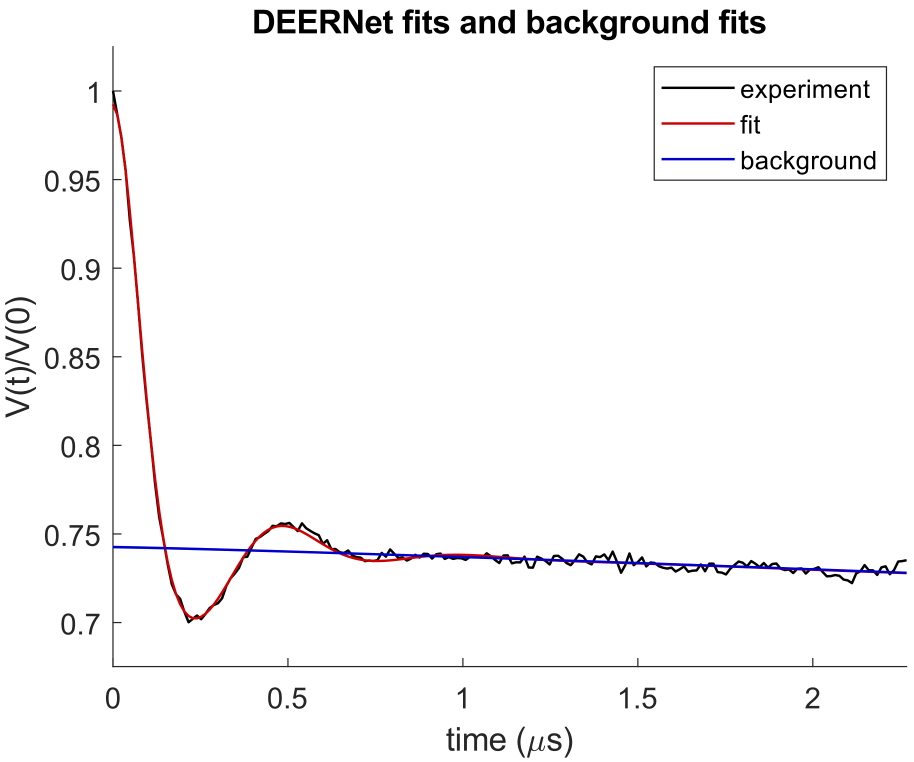

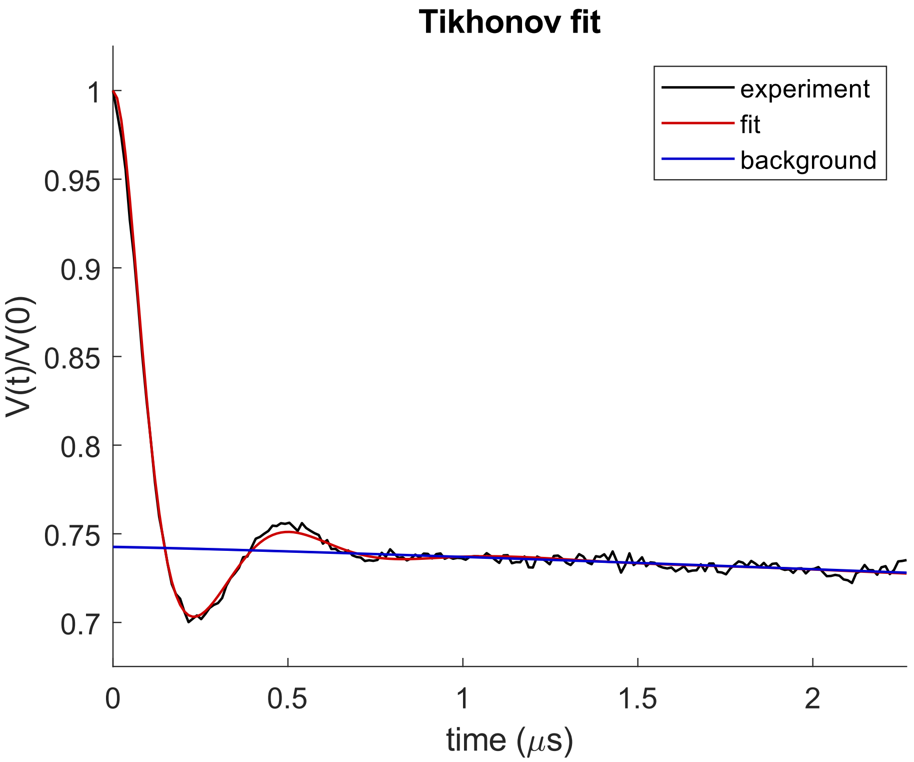

3. Experimental and processing parameters

Time increment: 12 ns

Maximum time: 2.268000e+03 ns

Zero time: 114 ns

Phase: -6.3 degree

Noise estimates normalized to maximum signal

From imaginary part: 0.00219

From DEERNet fit: 0.00254

From Tikhonov fit: 0.00304

Modulation depth: 0.257

Signal-to-noise ratio: 101.4 (w.r.t. modulation)

Ensemble of 32 neural networks

Background separation by neural network

Regularization parameter by best overlap with neural network solution

Regularization parameter used: 8.92

Reg. par. initial estimate by L-curve corner: 6.31

Overlap between DEERNet and regularization solutions: 0.908

Mean distance: 27.6 Å

Distance standard deviation: 2.4 Å

Full data set in Matlab format: 200223_BEBQ40.4_PELDOR_consensus_DEER_analysis.mat

Distance distributions in text format: 200223_BEBQ40.4_PELDOR_consensus_DEER_distribution.dat

Fit and background in text format: 200223_BEBQ40.4_PELDOR_consensus_DEER_fit.dat

DEER analysis report on dataset 200223_BEBQ40.16_PELDOR

DEERNet Spinach SVN Rev 5662 and DeerLab 0.9.1 Tikhonov regularization

ComparativeDEERAnalyzer

see: S. G. Worswick et al., DOI: 10.1126/sciadv.aat5218, L. Fabregas Ibanez et al., DOI: 10.5194/mr-1-209-2020

25-Oct-2021 11:48:03

1. Distance distributions

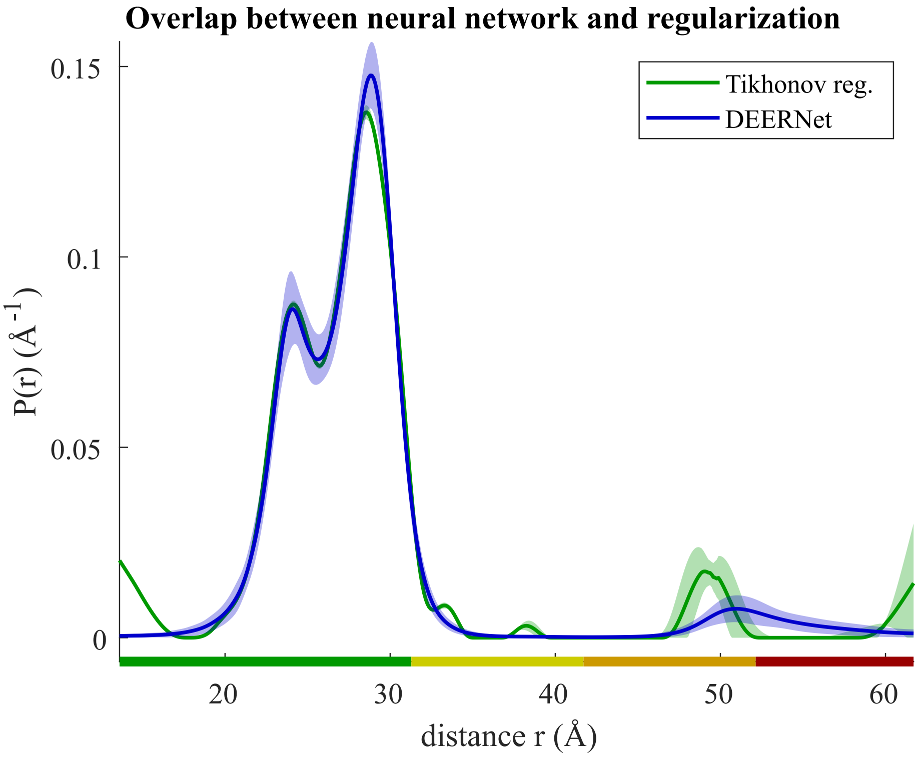

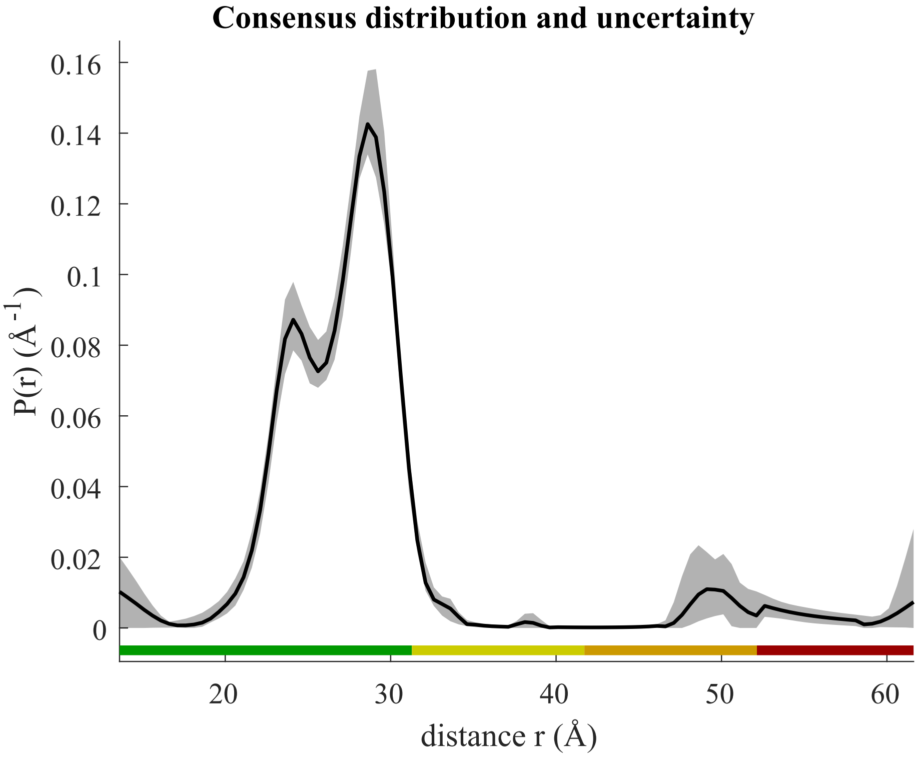

2. Fits of time-domain data

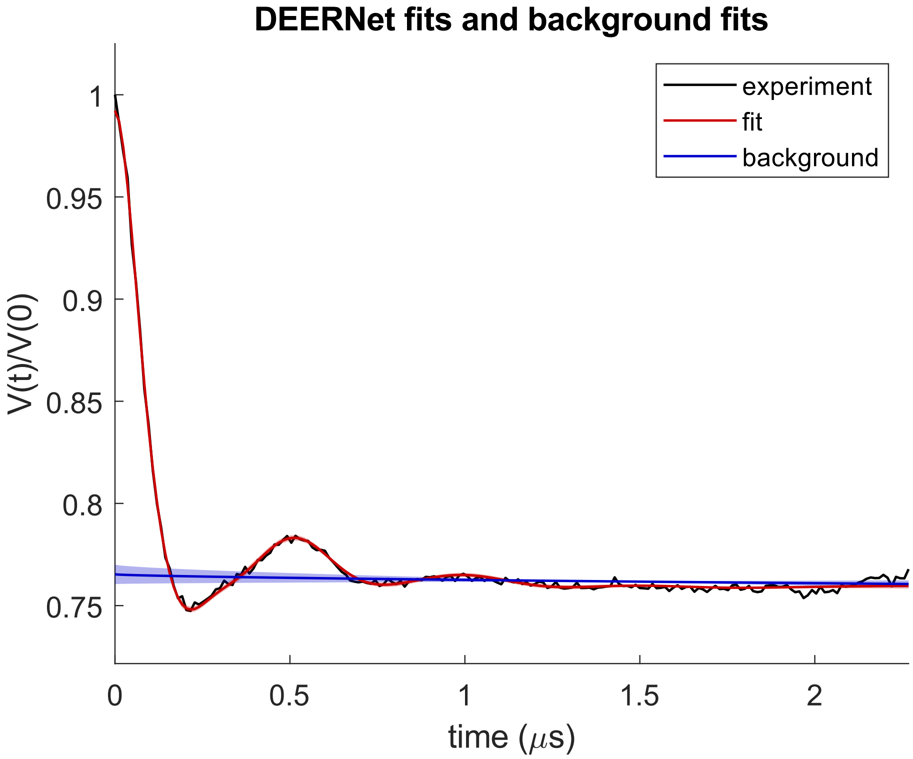

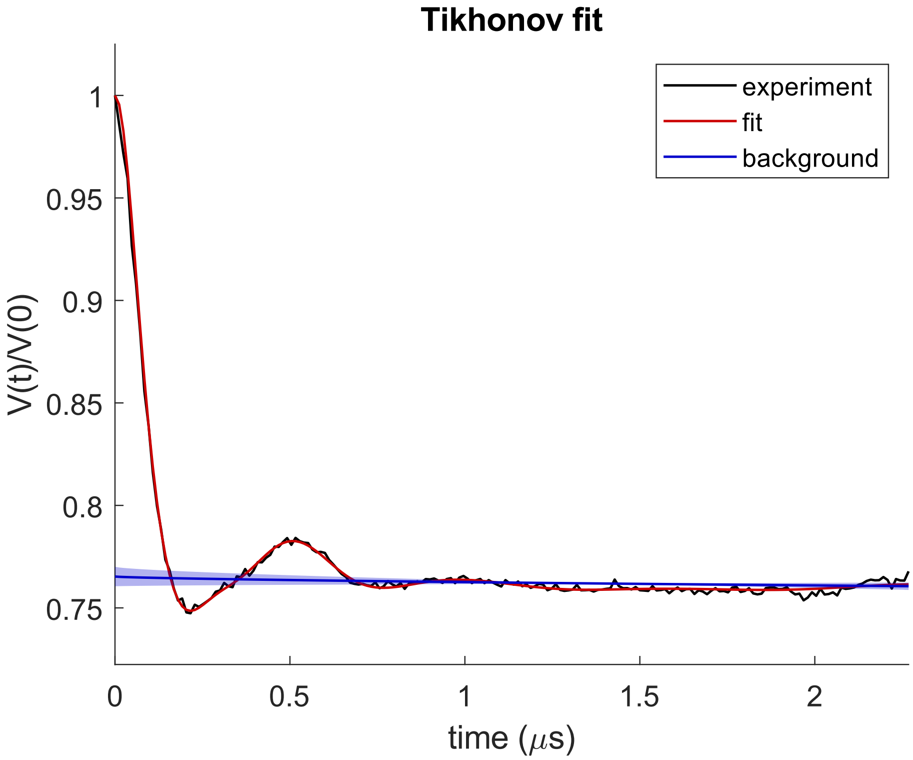

3. Experimental and processing parameters

Time increment: 12 ns

Maximum time: 2.268000e+03 ns

Zero time: 113 ns

Phase: -2.2 degree

Noise estimates normalized to maximum signal

From imaginary part: 0.00176

From DEERNet fit: 0.00200

From Tikhonov fit: 0.00224

Modulation depth: 0.235

Signal-to-noise ratio: 117.4 (w.r.t. modulation)

Ensemble of 32 neural networks

Background separation by neural network

Regularization parameter by best overlap with neural network solution

Regularization parameter used: 1.25

Reg. par. initial estimate by L-curve corner: 5.01

Overlap between DEERNet and regularization solutions: 0.915

Mean distance: 26.9 Å

Distance standard deviation: 3.0 Å

Full data set in Matlab format: 200223_BEBQ40.16_PELDOR_consensus_DEER_analysis.mat

Distance distributions in text format:
200223_BEBQ40.16_PELDOR_consensus_DEER_distribution.dat

Fit and background in text format: 200223_BEBQ40.16_PELDOR_consensus_DEER_fit.dat
